# Management of Chronic Graft-vs.-Host Disease in Children and Adolescents With ALL: Present Status and Model for a Personalised Management Plan

**DOI:** 10.3389/fped.2022.808103

**Published:** 2022-02-18

**Authors:** Agnieszka Sobkowiak-Sobierajska, Caroline Lindemans, Tomas Sykora, Jacek Wachowiak, Jean-Hugues Dalle, Halvard Bonig, Andrew Gennery, Anita Lawitschka

**Affiliations:** ^1^Department of Pediatric Oncology, Hematology and Transplantology, Poznan University of Medical Sciences, Poznan, Poland; ^2^Department of Pediatrics, Wilhelmina Children's Hospital, University Medical Center Utrecht, Utrecht University, Utrecht, Netherlands; ^3^Pediatric Blood and Bone Marrow Transplantation, Princess Máxima Center, Utrecht, Netherlands; ^4^Department of Pediatric Hematology and Oncology – Haematopoietic Stem Cell Transplantation Unit, National Institute of Children's Diseases and Medical Faculty, Comenius University, Bratislava, Slovakia; ^5^Hematology and Immunology Department, Robert-Debré Hospital, Assistance Publique-Hôpitaux de Paris and University of Paris, Paris, France; ^6^Goethe University Medical Center, Institute of Transfusion Medicine and Immunohematology, and German Red Cross Blood Center Frankfurt, Frankfurt, Germany; ^7^Medical School, Institute of Cellular Medicine, Newcastle University, Newcastle upon Tyne, United Kingdom; ^8^Stem Cell Transplantation Unit, St. Anna Children's Hospital, Medical University Vienna, Vienna, Austria; ^9^St. Anna Children's Cancer Research Institute, Vienna, Austria

**Keywords:** haematopoietic stem cell transplantation, chronic graft-vs.-host disease, paediatric, adolescent, management

## Abstract

Herein we review current practice regarding the management of chronic graft-vs.-host disease (cGvHD) in paediatric patients after allogeneic haematopoietic stem cell transplantation (HSCT) for acute lymphoblastic leukaemia (ALL). Topics covered include: (i) the epidemiology of cGvHD; (ii) an overview of advances in our understanding cGvHD pathogenesis; (iii) current knowledge regarding risk factors for cGvHD and prevention strategies complemented by biomarkers; (iii) the paediatric aspects of the 2014 National Institutes for Health-defined diagnosis and grading of cGvHD; and (iv) current options for cGvHD treatment. We cover topical therapy and newly approved tyrosine kinase inhibitors, emphasising the use of immunomodulatory approaches in the context of the delicate counterbalance between immunosuppression and immune reconstitution as well as risks of relapse and infectious complications. We examine real-world approaches of response assessment and tapering schedules of treatment. Furthermore, we report on the optimal timepoints for therapeutic interventions and changes in relation to immune reconstitution and risk of relapse/infection. Additionally, we review the different options for anti-infectious prophylaxis. Finally, we put forth a theory of a holistic view of paediatric cGvHD and its associated manifestations and propose a checklist for individualised risk evaluation with aggregated considerations including site-specific cGvHD evaluation with attention to each individual's GvHD history, previous medical history, comorbidities, and personal tolerance and psychosocial circumstances. To complement this checklist, we present a treatment algorithm using representative patients to inform the personalised management plans for patients with cGvHD after HSCT for ALL who are at high risk of relapse.

## Introduction

Allogeneic haematopoietic stem cell transplantation (HSCT) is a curative treatment for an increasing number of children and adolescents with various malignant and non-malignant haematological diseases, due to improved transplant procedures and reduced early mortality. However, successful long-term outcomes can be limited by chronic graft-vs.-host disease (cGvHD), which is often severe and is the most common complication post HSCT (1). This complex immune disorder resembles multiorgan autoimmune diseases and can result in adverse psychomotor development, functional impairment, disability and poor quality of life (2). It is believed to correlate with the graft- vs.-leukaemia effect (GvL) and contributes to a lower risk of relapse of malignancy (3). Moderate-to-severe cGvHD is the major cause of treatment-related mortality (TRM) and inferior overall survival (OS) following HSCT, and little progress has been made in recent decades regarding outcomes (4).

The publication of the National Institutes for Health (NIH) consensus criteria for cGvHD diagnosis and grading for use in clinical trials in 2005, as revised in 2014, represented a major advancement in the field (5, 6). Correspondingly, the German-Austrian-Swiss GvHD Consortium published a number of expert recommendations for daily clinical practice, including some considerations for the paediatric population (7). Recently, our understanding of cGvHD pathogenesis has improved substantially (8–10). The 2020 NIH Consensus Project has published documents aiming to move the field forward by summarising current knowledge and expert opinion, identifying the unmet needs of clinical care and gaps of knowledge, and outlining future research efforts (11–13).

Unfortunately, cGvHD in children and adolescents has been relatively understudied compared with in adults. Paediatric data on cGvHD pathophysiology, clinical manifestations, diagnosis and outcome are scarce. Furthermore, the NIH consensus criteria were primarily developed from adult data: their validation and clinical applicability for use in paediatric populations have been rarely investigated since their publication (14).

After HSCT to cure acute lymphoblastic leukaemia (ALL), paediatric patients are at high risk of developing various long-term sequelae, with cGvHD being one of the major risk factors (15). In view of the now longer life expectancy of children post HSCT and the significant cGvHD-related morbidities within a growing body, better understanding and management of paediatric cGvHD is imperative.

To aid this, we herein review the current knowledge regarding the management of paediatric cGvHD. Specific topics covered include: the epidemiology and pathogenesis of cGvHD, risk factors, biomarkers and paediatric aspects of the 2014 NIH criteria for diagnosis and grading. Furthermore, we present current options for treatment, with emphasis on topical therapy, immunomodulatory interventions and supportive care and with consideration for the delicate counterbalance between immunosuppression and immune reconstitution, risk of relapse and risk of infectious complications. We aim to present a new perspective on how management strategies can be tailored to the specific needs of individual patients and provide a framework for the personalised treatment of paediatric patients with cGvHD after HSCT for ALL to support clinicians in daily clinical practice.

### Methods

We searched PubMed to find English-language articles from 1970 to 2021 emphasising on paediatric data whenever possible. We used the following terms: “chronic GVHD with and without paediatric/children,” “pathogenesis, pathophysiology,” “epidemiology, incidence,” “diagnosis and grading,” “risk factors,” “biomarker,” “immune reconstitution,” “treatment,” “management,” “topical treatment,” “ECP,” “MSC,” “supportive and ancillary care,” “relapse,” and “infections and infectious complications.” The reference lists in the selected studies were reviewed to identify additional articles. No limits were applied in the initial search, but we then excluded articles that contained only adult case series focusing on experimental approaches.

## Current Knowledge and Practice

### Epidemiology of cGvHD in Children and Adolescents

Nowadays, the criteria for diagnosis of cGvHD are based on the combination of clinical manifestations and time of onset according to the NIH consensus criteria (5). This should be kept in mind for comparison of published data on the incidence of acute GvHD (aGvHD) and cGvHD.

The incidence of paediatric cGvHD shows great variety (ranging from 6 to 65%), with some differences explained by the specific transplant indication (malignant vs. non-malignant), heterogeneity of transplant procedures, and age-related immune reconstitution post transplantation (16). In general, paediatric cGvHD tends to be less common and somewhat milder than cGvHD in adults (17–19).

Stem cell source can influence risk of GvHD. The lowest incidence of cGvHD (6%) was observed among paediatric patients undergoing cord blood HSCT (20, 21). In historical data from the 1990s and early 2000s, the incidence of paediatric cGvHD after HSCT for haematological malignancies ranged from 28% with a sibling donor to 52–65% with an unrelated donor. The incidence and severity of cGvHD was higher in patients after peripheral blood stem cell (PBSC) HSCT than after bone marrow (BM) HSCT (22, 23).

Underlying disease and age can also affect cGvHD risk. Zecca et al. reported in 2002 a higher incidence of cGvHD in patients with malignant (35%) vs. non-malignant (13%) diseases in a retrospective analysis of 696 children. Furthermore, the lowest incidence of cGvHD (9%) was described for children <2 years of age, and the highest (44%) for patients >15 years of age (24). Likewise, Qayed et al. found in a retrospective analysis of 476 paediatric ALL patients after matched sibling donor (MSD) BM HSCT during the years 2000 to 2013 a cumulative incidence of cGvHD of 16%; a lower risk of cGvHD was observed for the age group 2 −12 years in comparison to patients >12 years old (25). A retrospective, single-centre analysis of 146 children with malignant and non-malignant diseases transplanted at the St. Anna Children's Hospital, Vienna, between 2004 and 2012 revealed a cumulative incidence of reclassified NIH-defined cGvHD (2005 criteria) of 18% and 21% at 1 and 3 years post HSCT, respectively. A multivariate analysis identified donor age >5 years as risk factor for the development of cGvHD but there was no association between recipient age and cGvHD risk (Lawitschka et al., unpublished data). One of the most recent prospective multicentre studies of paediatric cGvHD, by Cuvelier et al., indicated an incidence of 21% of accurately assessed NIH-defined cGvHD in 243 paediatric patients with various malignant and non-malignant diseases undergoing a range of transplant procedures. Recipients ≥12 years of age were at higher risk for cGvHD in comparison to younger patients, and *de novo* cGvHD occurred almost exclusively in patients ≥12 years, indicating a crucial role of aGvHD in the pathogenesis of cGvHD in infants (14).

The overall incidence of cGvHD in paediatric patients has decreased over recent decades. This is contrary to the pattern observed in adult studies, probably due to the widespread use of granulocyte colony-stimulating factor (G-CSF)–mobilised PBSCs over BM grafts in adults (26). The older age of transplanted patients, and the use of reduced intensity regimens which require GvL effect also contribute to the higher incidence of cGvHD in this group (27, 28).

### Pathogenesis of cGvHD in Children and Adolescents

The immunobiology of cGvHD differs distinctly from that of aGvHD (29). Despite major advances in the field, the complex and multifactorial pathogenesis of cGvHD is not fully understood and incorporates failure of central and/or peripheral tolerance mechanisms in the presence of minor (and major) major histocompatibility complex (MHC) polymorphisms (30). It is well-known that cGvHD is a pleomorphic syndrome resembling many autoimmune diseases but, in addition, appreciation of its correlation with monogenic immune disorders may lead to better understanding of its pathogenesis, especially in paediatric populations (31).

Cooke et al. (8) has proposed a triphasic model of cGvHD pathogenesis which involves: (i) acute inflammation with tissue injury and vascular inflammation (which may be subclinical); (ii) dysregulated immunity, thymic damage and dysfunction with the transition to chronic inflammation; followed by (iii) dysfunctional tissue repair resulting in the deposition of collagen and development of fibrosis. Recently, major advances in cGvHD research have been made but these are largely based on murine models that do not reflect the whole clinical spectrum of human cGvHD (10).

In general, a complex cytokine-driven cellular network (32) involving damage of the thymus and germinal centres with aberrant interactions between donor-derived subsets of effector T and B cells contributes to both the immune pathology of cGvHD and innate immune responses with unusual antigen presentation. Of note, multiple pathogenic pathways may operate simultaneously.

Regarding the T-cell compartment, various models have demonstrated a critical role of naïve T cells with further dysregulation of CD4^+^ T helper (Th17), CD8^+^ T cell (Tc17) and T-follicular helper (Tfh) cell differentiation (32, 33) together with reduced numbers of regulatory T cells (T_reg_) (34). High interleukin (IL)-6 levels after HSCT lead to IL-17–secreting Th17 and Tc17 differentiation (35, 36). This process is augmented by stem cell mobilisation with G-CSF. Th17/Tc17 produce multiple cytokines, including interferon gamma (IFN-γ), tumour necrosis factor (TNF), IL-22, colony-stimulating factor-1 (CSF-1), and granulocyte-macrophage colony-stimulating factor (GM-CSF) which promote the migration and differentiation of monocytes into pathogenic macrophages. Simultaneously, Tfh produce IL-21 which is critical for germinal centre B-cell formation and antibody secretion (both autoreactive and alloreactive) (37).

Regarding the B-cell compartment, an expansion of germinal centre B cells with subsequent allo/autoantibody secretion has been shown (38). B cells of cGvHD patients have increased survival capacity and signal through B-cell activating factor (BAFF) and B-cell receptor (BCR) signalling pathways. BAFF is produced primarily by myeloid cells, stromal cells and some lymphoid cells. BAFF:B-cell ratios are elevated in patients with active cGvHD (39). The BCR-signalling molecules Syk and Bruton tyrosine kinase (BTK) appear to be hyper-activated in B cells during cGvHD (37).

Additionally, a role of the gut microbiome has been observed in cGvHD, with the loss of flora diversity after HSCT recently reported to correlate with inferior outcome (an increased risk of mortality) (40, 41).

In the context of the transition to fibrosis, the involvement of macrophages producing the profibrotic cytokines tumour growth factor beta (TGF-β) and platelet-derived growth factor alpha (PDGF-α) leads to the deposition of collagen secreted from activated fibroblasts (42).

Better understanding of the pathogenic pathways of cGvHD is being translated into the clinic in the form of rationales for specific treatment schedules. This may pave the way for novel promising therapeutic approaches that potentially target various cytokines, cell subsets and signalling pathways (30). Furthermore, it serves as a basis for more individualised treatment plans in cGvHD (10).

### Biomarkers for Paediatric cGvHD

The multisystemic, polymorphic nature of cGvHD and challenges in clinical diagnosis such as lung involvement in infants (14) makes the identification of potential GvHD biomarkers of utmost importance. Biomarkers are defined as biochemical or cellular variables categorised according to how they are used. Three subtypes of biomarker in cGvHD are recognised: (i) diagnostic biomarkers used to identify GvHD patients at the onset of the disease and to aid differential diagnosis; (ii) prognostic biomarkers used to identify patients with different degrees of risk for GvHD occurrence, progression or resolution before the onset of clinical cGvHD manifestation of the disease; and (iii) predictive biomarkers used to categorise patients based on their likelihood to respond to therapy (43, 44).

Great effort has been put into identifying relevant cGvHD biomarkers. It is important to keep in mind that patients with cGvHD represent a heterogeneous group with various characteristics having only the diagnosis but not the phenotype in common. Variables such as age, primary disease for which HSCT was indicated, treatment modalities and transplant procedures, and post-transplant complications have a great impact on immune reconstitution (45) and may influence the biomarkers present (10, 44).

Among those considered to be validated plasma biomarkers are soluble BAFF, a panel consisting of 4 biomarkers [ST2, chemokine (C-X-C) motif ligand 9 (CXCL9), matrix metalloproteinase 3 (MMP-3) and osteopontin], CXCL10, and chemokine (C-C motif) ligand 15 (CCL15) (Table 1) (46–63). Validated cellular biomarkers include CD163, B cells expressing toll-like receptor 9 (TLR9), B cells defined as CD19^+^/CD21^low^ B cells, a high BAFF:B cell ratio in the plasma, T_regs_, CD4^+^CD146^+^CCR5^+^ T cells and Tfh cells (Table 1) (64). We briefly discuss these biomarkers below.

**Table 1:** Validated Biomarkers in cGvHD.

**Biomarker**		**References**	**Age range, years**	**Association with cGvHD**	**Use**
Plasma	sBAFF	(46)	1–29	↑	Diagnostic
		(47)	21–68	↑	Diagnostic
		(48)	18–68	↑	Diagnostic/prognostic
	4 biomarker panel (ST2, CXCL9, MMP-3, and osteopontin)	(49)	1–79	↑	Diagnostic/prognostic
	CXCL9	(49)	13–59	↑	Diagnostic
		(50)	0–79	↑	Diagnostic
	CXCL9, CXCL10	(51)	21–68	↑	Diagnostic
	CXCL10	(52)	≤ 18	↑	Diagnostic
	CCL15	(63)	19–79	↑	Diagnostic/prognostic
	MMP-3	(53)	19–73	↑	Diagnostic
Cellular	CD163	(54)	19–73	↑	Diagnostic
	TLR9^+^ B cells	(55)	1–29.9	↑	Diagnostic
	CD21^low^ B cells	(56)	20–66	↑	Diagnostic
	sBAFF:B cell ratio	(47)	19–66	↑	Diagnostic
		(57)	23–59	↑	Diagnostic
	T_regs_	(58)	NR	↓	Diagnostic
	CD4^+^CD146^+^CCR5^+^ T cells	(59)	25.9–75.6	↑	Diagnostic
	Tfh cells	(60)	25–75.6	↓	Diagnostic

↑ *, increased in cGvHD*;

↓ *, decreased in cGvHD; CCL15, chemokine (C-C motif) ligand 15; cGvHD, chronic graft- vs.-host disease; CXCL, chemokine [C-X-C] motif ligand; MMP-3, matrix metalloproteinase 3; NR, not reported; sBAFF, soluble B-cell activating factor; Tfh, T follicular helper; TLR9, toll-like receptor 9; T_regs_, regulatory T cells*.

#### Plasma Biomarkers

##### Soluble B-Cell Activating Factor

High levels of sBAFF have been found in patients with active cGvHD and have been linked with both early onset (3–8 months) and late onset (≥9 months) disease (46, 47). A significant decrease in sBAFF was found in responders to corticosteroids 2 months after their initiation (46). Moreover, Saliba et al. (47) described increased sBAFF levels at the time of diagnosis of cGvHD as a potential predictor of non-relapse mortality (NRM) (48). Because of its significant presence in various settings of cGvHD, sBAFF is described as both a diagnostic and prognostic biomarker (64). One major limitation is the steroid sensitivity of sBAFF, which becomes undetectable on steroid doses >0.5 mg/kg prednisolone independent of response to treatment.

##### A Panel of ST2, CXCL9, MMP-3 and Osteopontin

In a study of Yu et al. (61), a panel of 4 proteins (ST2, CXCL9, MMP-3, and osteopontin) was found to significantly correlate with cGvHD diagnosis, cGvHD severity and NRM. When measured at day +100, the panel could predict cGvHD occurring within the next 3 months, even in the absence of known clinical risk factors. In addition, increased MMP-3 is associated with the development of bronchiolitis obliterans (50). Solely elevated plasma concentrations of CXCL9 are considered to be an independent cGvHD biomarker (49, 50).

##### CXCL10 and CCL15

Similarly to CXCL9, CXCL10 is an inflammatory chemokine involved in the activation and recruitment of T cells, eosinophils, monocytes and natural killer (NK) cells. In a study by Kariminia et al. (52), CXCL10 met the criteria for replication as a clinical biomarker for the diagnosis of cGvHD. Although plasma concentrations of CCL15 were found to be elevated in cGvHD patients compared with controls and were associated with NRM, levels at day +100 could not predict cGvHD occurring within the next 3 months with clinically relevant sensitivity/specificity (63).

#### Cellular Markers

In a study of Inamoto et al. (54), a higher cellular expression of CD163 at day +80 was associated with *de novo* cGvHD. CD163—a macrophage receptor—is expressed at increased levels during oxidative stress; therefore, the authors concluded that monocyte or macrophage activation may contribute to the pathogenesis of cGvHD.

Sarantopoulos and colleagues in 2009 suggested that B cells play a role in cGvHD pathogenesis through the presence of alloantibodies and high plasma sBAFF levels: both are found in patients with cGvHD. Detailed phenotypic and functional analyses of peripheral B cells in patients after HSCT showed that, in patients with cGvHD, significantly higher BAFF:B cell ratios are observed compared with patients without cGvHD or with healthy donors (38, 47). Other B cell subsets associated with the development of cGvHD are those that express TLR9 (55) and CD21^low^ B cells (56).

Tfh cells play an important role in the regulation of B cell immunity. Extensive phenotypic and functional analyses of circulating Tfh cells demonstrated that patients with active cGvHD had a significantly lower frequency of circulating Tfh cells compared with patients without cGvHD (60).

CD4^+^CD146^+^CCR5^+^ T cells are frequent in cGvHD patients. According to Forcade et al. (59), these cells proved to be sensitive to pharmacological inhibition (59).

Zorn et al. (58) conducted a phenotypic study of T_regs_ and demonstrated a decreased frequency in patients with cGvHD compared with patients without cGvHD (*p* < 0.001) and healthy individuals. A different study has connected an increased Th17:T_reg_ ratio to the development of liver cGvHD (65). Moreover, Alho et al. (66) confirmed a decreased frequency of T_regs_ and shortened T_regs_ telomeres in patients with cGvHD.

#### cGvHD Biomarkers in Children: Children Are Not Small Adults

It is known that children have a lower rate and perhaps different presentation of cGvHD compared to that seen in adults (25). One of important aspects of cGvHD pathophysiology is the variability of immune reconstitution between patients after HSCT, which is age related and dependent on thymic hormones (as described in a companion article by Eyrich et al. in this supplement of Frontiers in Paediatrics). Therefore, it is important to determine differences among cGvHD biomarkers in adult and paediatric populations.

Few studies have investigated age-related differences in the biology of cGvHD (16, 67). Recently, Lawitschka et al. (45) demonstrated for the first time in a highly homogenous paediatric patient cohort that both cGvHD and its activity were associated with the perturbation of the B cell compartment, including low frequencies of CD19^+^CD27^+^ memory B cells and increased frequencies of circulating CD19^+^CD21^low^ B cells. The immunological profile of patients with cGvHD in a paediatric cohort studied by Schultz et al. (67) had distinctive features, with increased activated T cells, naïve Th cells and cytotoxic T cells, loss of CD56^bright^ regulatory NK cells, and increased levels of ST2 and soluble CD13. When cohorts of adults and children who had undergone HSCT were compared, significant differences were found (16). Elevated levels of ST2 and naïve Th cells, and depression of NK regulatory cells were present in both children and adults. However, children presented with broad suppression of newly formed B cells whereas adults demonstrated increased T1-CD21^low^ B cells and decreased T1-CD24^high^CD38^high^ B cells. T_reg_ abnormalities in children were primarily present in memory T_regs_, whereas in adults the abnormalities were in naïve T_regs_. Aminopeptidase N (sCD13) and intercellular adhesion molecule 1 (ICAM-1) were significantly increased only in prepubertal children with cGvHD (16). The authors concluded that the recipient's age at the time of HSCT impacts on the immune profile of cell populations and cytokines occurring in cGvHD.

Even though there are several validated biomarkers for cGvHD, studies that associate biomarkers with severity, activity and resolution of the disease are lacking. Studies with mixed age cohorts may show trends, but immune reconstitution is age related and this needs to be taken into consideration when evaluating biomarkers and the pathophysiology of cGvHD. The verification and validation of candidate biomarkers in paediatric populations is highly relevant since this is a notoriously underrepresented population within clinical trials and adult data may not be extrapolated to the paediatric population (44). Thus, more age-specific studies of biomarkers are needed because children are simply not “small adults.”

### Risk Factors for the Development of cGvHD and Prevention Strategies

Since cGvHD is a highly polymorphic complication of HSCT, much clinical research has been done to characterise disease severity at onset and to define risk factors for the development of cGvHD and for predicting poor survival (1, 68). However, published data on risk factors for paediatric cGvHD often stem from combined adult and paediatric studies and are mutually incomparable because important details of patient and transplant characteristics are incomplete, such as use of conventional vs. high-resolution human leukocyte antigen (HLA) disparity, details of GvHD prophylaxis including blood concentrations and duration of given agents, kinetics of engraftment and chimerism with imminent relapse, and antigenaemia and infections.

Below we provide an overview of risk factors for the occurrence of cGvHD (Table 2) (14, 17, 24, 25, 27, 69–82) and prognostic factors associated with poor outcomes in patients with cGvHD (Table 3) (75, 76, 81, 83–99), prioritising paediatric data wherever possible.

**Table 2:** Summary of risk factors for cGvHD identified in studies, with an emphasis of paediatric cohorts.

**References**	**Patients,** ***N***	**Study type**	**Risk factors**																	
			**UD**	**MMD**	**PBSC**	**Female donor/male recipient**	**Parity of female donor**	**Older donor age**	**Older recipient age**	**Malignancy**	**CMV+**	**RIC**	**TBI**	**Radiation**	**Busulfan**	**No TCD**	**GVHD Px not CsA + MTX**	**Amount of CD19^**+**^ CD3^**+**^ cells infused**	**Prior aGvHD**	**Cohort age, median/mean (range)**
Zecca et al. (24)	696	MC															✓			Median 7 yr (0.3–17)
Diaz et al. (69)	80																✓			Mean 13 yr (1–18)
Eapen et al. (70)	773	MC			✓															Median 17 yr (8–20)
Ozawa et al. (71)	2,937	MC				✓		✓	✓				✓						✓	Median 27 yr (0–67)
Williams et al. (72)	case report/review	CR													✓ BOS					NR
Baird et al. (17)	Review	MC	✓	✓		✓		✓	✓	✓			✓						✓	NR
Flowers et al. (71)	2941	SC	✓	✓	✓	✓	✓	✓	✓											Median 40.3 yr (0.6–71.6)
Lee et al. (73)	23	SC																	✓	Mean 12 yr (1–18)
Kanda et al. (74)	4,818	MC			✓	✓					✓				✓	✓			✓ Gr 2–4 aGvHD	(16–82 yr)
Arai et al. (75)	26,563	MC	✓		✓ PBSC + BM	✓			✓											(1–79 yr)
Grube et al. (76)	243	SC	✓ MMUD		✓														✓ Gr 3–4 aGvHD	Mean 48 yr (16–71)
Lazaryan et al. (77)	469	SC			✓				✓											(0–74 yr)
Watkins et al. (78)	442	SC						✓	✓											Median 12 yr (0.6–21)
Afram et al. (27)	820	MC				✓severe cGvHD			✓										✓	(1–70 yr)
Qayed et al. (25)	476	MC						✓	✓≥13 yr								✓			(1–17 yr)
Cuvelier et al. (14)	243	MC			✓				✓≥13 yr									✓	✓ Gr 2–4 aGvHD	(0.2–18 yr)
Kok et al. (79)	98	SC																✓		NR
For sclerotic cGvHD:																				
Martires et al. (80)	206	SC												✓	✓	✓			✓	NR
Inamoto et al. (81)	977	SC													✓	✓			✓	Median 48 yr (0–78)

*✓, associated with the risk of cGvHD; A, adult; BOS, bronchiolitis obliterans syndrome; Ad, adolescent patients; CMV+, cytomegalovirus seropositivity; Gr, grade; MC, multicentre; MMD, mismatched donor; MMUD, mismatched unrelated donor; NR, not reported; P, paediatric; PBSC, peripheral blood stem cell; RIC, reduced-intensity conditioning; SC, single centre; TBI, total body irradiation; TCD, T-cell depletion; UD, unrelated donor; yr, years*.

**Table 3:** Summary of risk factors for higher NRM and lower OS in patients with cGvHD identified in studies.

**References**	**Patients, N**	**Study type**	**Cohort mean/median age (range)**	**Risk factors**																									
				**Karnofsky/lansky performance scale**	**Older patient age**	**HLA mismatch**	**Disease status**	**Donor type (MMD)**	**Sex mismatch (donor/recipient)**	**PBSC**	**Progressive cGvHD**	**Lichenoid skin**	**Hyperbilirubinaemia**	**Thrombocytopenia**	**Eosinophilia**	**Low eosinophils**	**LALC**	**GI tract involved/diarrhoea**	**Steroid use/dose**	**Prior aGvHD**	**Time to cGvHD**	**GvHD prophylaxis**	**NIH global severity**	**NIH organ severity**	**NIH lung score**	**NIH skin score**	**Signs of aGvHD (NIH)**	**Overlap cGvHD (NIH)**	**Outcome measure**
Jagasia et al. (83)	110	MC	P/A mean 42 yr (1–65)																								✓		OS
Pérez-Simón et al. (84)	171	SC	Ad/A mean 45 yr (14–69)								✓												✓						OS
Cho et al. (85)	211	SC	Ad/A mean 34 yr (15–60)																				✓						OS
Vigorito et al. (86)	740	SC	P/A (0.8–67)		✓	✓			✓				✓	✓					✓	✓									NRM
Kim et al. (87)	196	SC	P/A (10–59)	✓		✓	✓	✓ UD												✓			✓						NRM/OS
Pidala et al. (88)	427	MC	A (NR)	✓										✓									✓					✓	NRM/OS
Arai et al. (75)	298	MC	A (19–78)	✓										✓									✓						NRM/OS
Arora et al. (89)	5,343	MC	P/A mean 36 yr (<1–72)	✓	✓		✓	✓ MMD	✓				✓	✓						✓	✓	✓							NRM/OS CIBMTR risk score: 6 risk groups
Pérez-Simón et al. (90)	336	MC	P/A mean 50 yr (1–69)	✓										✓			✓	✓					✓						NRM/OS
Jacobsohn et al. (91)	1,117	MC	P mean 12 yr (<1–19)	✓	✓	✓✓	✓			✓				✓															NRM/OS
Jacobsohn et al. (92)	458	MC	P >2 yr																							✓			NRM/OS
Tecchio et al. (93)	159	SC	NR		✓		✓																						NRM
Baird et al. (94)	189	MC	A (NR)												✓										✓				OS
Inamoto et al. (81, 95)	376	MC	A (NR)	✓	✓		✓	✓ MMD	✓				✓	✓						✓	✓	✓							NRM/OS
Inamoto et al. (95)	574	MC	A (19–79)																				✓	✓					NRM/OS
Moon et al. (96)	346	SC	A mean 46 yr (18–70)	✓					✓					✓						✓			✓						NRM/OS
Palmer et al. (97)	496	MC	P/A ≥2 yr																						✓				NRM/OS
Ayuk et al. (98)	201	SC	A median 54 yr (18–75)						✓					✓															NRM/OS
Grube et al. (76)	243	SC	Ad/A mean 48 yr (16–69)								✓			✓									✓						NRM/OS
Moon et al. (99)	307	SC	A median 46 yr (18–70)	✓	✓		✓	✓ MMD	✓				✓	✓		✓	✓			✓	✓	✓							OS, revised CIBMTR risk score

*A, adult; Ad, adolescent; aGvHD, acute graft- vs.-host disease; cGvHD, chronic graft- vs.-host disease; CIBMTR, Centre for International Blood and Marrow Transplant Research; GI, gastrointestinal; HLA, human leukocyte antigen; LALC, lower absolute lymphocyte count; MC, multicentre; MMD, mismatched donor; NIH, National Institutes for Health; NR, not reported; NRM, non-relapse mortality; OS, overall survival; P, paediatric; PBSC, peripheral blood stem cell; SC, single centre; UD, unrelated donor; yr, year*.

#### Risk Factors for the Development of cGvHD

The following risk factors for cGvHD post HSCT have been published and summarised in reviews and recommendations: preceding aGvHD, the use of an unrelated donor or mismatched donor, PBSCs as the donor source, older (≥12 years) recipient or donor age, female donor for a male recipient, parity of female donor, malignant primary disease and the use of total body irradiation (TBI) (Table 2).

By far the most powerful predictor for the development of cGvHD seems the severity of aGvHD (17, 24, 83). A lower incidence and severity of aGvHD and cGvHD has been associated with the use of *ex vivo* or *in vivo* T-cell depletion (TCD). However, use of TCD poses a risk of graft failure, infection and relapse (12); further data in paediatric HSCT for ALL are needed.

#### Prognostic Factors Associated With Higher NRM and/or Poorer OS

Regarding prognostic factors at the onset of cGvHD that are associated with increased mortality, the development, validation and the revision of the NIH consensus criteria for diagnosis and staging of cGvHD (5, 6) have moved the field forward substantially (see Supplementary Material). In children, Cuvelier et al. reported on the feasibility and reliability of the NIH consensus criteria and concluded that further refinement was needed (14). The NIH global severity score of cGvHD has been validated in various adult studies, but less so in children (84, 85, 87, 90) and adolescents (76). In a large paediatric Centre for International Blood and Marrow Transplant Research (CIBMTR) study including 1,117 patients, Jacobsohn et al. found the following variables to be associated with higher NRM in a multivariate analysis: mismatched unrelated donor (MMUD), PBSC as the stem cell source, Karnofsky/Lansky performance score <80, and platelet count <100 × 10^9^/L. Regarding worse OS, the study reported age >10 years, an MMUD, advanced disease at transplantation, Karnofsky/Lansky score <80; and platelet count <100 × 10^9^/L as significant risk factors (70). Additional risk factors associated with poor prognosis are direct progression from aGvHD to cGvHD and organ-specific aspects such as lung and gastrointestinal tract involvement and hyperbilirubinaemia (76) (Table 3).

Prior to publication of the NIH consensus criteria, a CIBMTR cGvHD risk score had been developed (89, 100). Studies in adults reported on improved prognostic stratification when combining the CIBMTR cGvHD score with the NIH criteria (95, 101).

To address the question of risk factors for cGvHD in paediatric patients, we studied retrospective data on 358 paediatric patients who underwent HSCT between 1980 and 2012 at the St. Anna Children's Hospital, Vienna, and who survived relapse-free beyond day +100. We identified in multivariate analyses older donor age (>5 years), prior aGvHD of grade 2–4, and a female donor for a male recipient as risk factors for the development of cGvHD. Overall mortality was significantly higher for patients >10 years old and for those with moderate-to-severe global severity score, while sclerotic cGvHD was independently associated with a lower risk of death (A. Lawitschka, unpublished data).

Within the NIH 2020 initiative a summary has been provided about the major advances in understanding of the aetiopathology of cGvHD and future efforts (11, 102, 103). The field is moving toward clinical studies targeting prevention strategies that decrease the risk of morbid cGvHD such as moderate-to-severe cGvHD without an increased risk of relapse or infection. Regardless of the incidence of cGvHD, morbid forms of cGvHD like fasciitis and lung GvHD lead to excess long-term morbidity and a future aim is to avoid these. Therefore, it is important to evaluate risk factors for the development and the outcome of cGvHD and to predict the highly morbid forms.

The 2020 NIH cGVHD consensus group agreed on the need for adoption of primary study endpoints measuring survival without moderate-to-severe cGvHD, such as cGvHD-free and relapse-free survival (CRFS). This remains challenging as studies need a minimum of 1 year of follow-up to assess relevant endpoints of cGvHD (12). In this regard, an important consideration for paediatric studies may be that endpoints should be tailored to non-malignant and malignant primary diseases separately because patient and HSCT characteristics, GvHD prophylaxis and treatment modalities differ distinctly between those patient groups (104).

### NIH-Defined Diagnosis, Organ Scoring and Staging of cGvHD

The 2005 NIH Consensus Conference proposed new criteria for diagnosing and scoring the severity of cGvHD in clinical trials (6). The 2014 NIH consensus maintained the prior framework but revised the criteria and provided guidelines for cGvHD definition, endpoint reporting and trial design. The main revisions were made for the subcategory of overlap cGvHD and the distinction between active disease and past tissue damage (5). Recently, a joint task force added some specifications to the NIH consensus criteria, with focus in associated manifestations and steroid sensitivity (105).

The 2014 NIH consensus criteria include clinical symptoms in 8 organs, laboratory findings and pulmonary function tests. Each organ is graded from 0 to 3; the overall severity is classified as mild, moderate or severe depending on the number of affected organs and the involvement severity. Symptoms can be stratified as diagnostic, distinctive and in common with aGvHD (5).

Patients who are lacking diagnostic signs of cGvHD require histological confirmation if new systemic immunosuppressive treatment is to be introduced, especially in the case of treatment failure. Exclusion of differential diagnoses such as infection is required (105). The most commonly affected organ is the skin, followed by the eyes (14, 73). Patients may show other immune-mediated manifestations also (termed “other, associated” manifestations), which should be evaluated although they do not contribute to grading. Regarding the type of onset of cGvHD, the following definitions are applied: progressive (progression from aGvHD without resolution), quiescent (prior aGvHD with resolution), and *de novo* (without any history of aGvHD) (105).

#### Applying and Adapting the NIH Criteria to Paediatric Patients

Originally, the NIH consensus criteria were not validated in patients under 18 years of age. Lee et al. attempted to implement the 2005 criteria in paediatric patients (73). Furthermore, a paediatric adaption has been developed by Lawitschka et al. (1), which has been revised for clinical use within the paediatric transplant centres of the German-Austrian-Swiss Consensus Group (www.GVHD.de), but as yet is not validated. In 2019, Cuvelier et al. (14) reported important data from a prospective multi-institutional study on biomarkers in cGvHD in 302 paediatric patients for which the 2014 NIH criteria were not only applied but also reviewed by a study adjudication committee. Although 28% of cGvHD cases were reclassified, the authors reported that the application of NIH criteria was feasible and reliable in a paediatric population. In that study cohort, the incidence of late acute and classic cGvHD was similar (25 vs. 21%, respectively), which underlines the relationship between aGvHD and cGvHD; in fact, very few children have true *de novo* cGvHD and aGvHD of grade 2–4 is one of the main risk factors for developing cGvHD (see Table 2). The NIH criteria have also been adjusted for paediatric patients for the diagnosis and staging of pulmonary GvHD (14).

### Treatment for Paediatric cGvHD

#### First-Line Classic Immunosuppressive Therapy

In mild cGvHD, patients may only require topical treatment depending on the organs involved and the risk of relapse of the underlying disease (106). In multiorgan involvement at cGvHD onset, moderate-to-severe or/and high-risk cGvHD (see section Risk Factors for the Development of cGvHD and Prevention Strategies on risk factors) immunosuppressive treatment is necessary. The recommended first-line immunosuppressive treatment comprises a corticosteroid (prednisone 1 mg/kg/day) with or without a calcineurin inhibitor (CNI) (107, 108), with topical therapy wherever possible; this applies to moderate and severe cGvHD at onset also (109). The addition of a CNI to corticosteroid therapy does not increase the response rate but allows for a reduction in corticosteroid dosing that can reduce long-term side effects. Koc et al. compared in a randomised study prednisone vs. prednisone plus cyclosporine in patients with cGvHD (*n* = 307; age 0.9–57.1 years) without thrombocytopenia and reported similar outcomes for both study groups, with the exception that steroid-associated toxicity was lower with prednisone plus cyclosporine (110).

Recently, rituximab was evaluated as a part of the first-line therapy of cGvHD. In a phase 2, prospective trial (*n* = 24 adults) it was added to a corticosteroid and CNI for newly diagnosed cGvHD (111). The overall response rate (ORR) at 1 year was 83% and the 1-year cumulative incidence of NRM was 14%. In two other studies on rituximab as the frontline therapy of cGvHD (112, 113), the cumulative incidence of cGvHD resolution at 3 years was 71–77% and the rate of NRM was 15–19%.

The efficacy of rituximab-based first-line treatment of cGvHD needs further investigation. In this regard, paediatric data are lacking. There is an ongoing clinical trial on the use of itacitinib and extracorporeal photopheresis (ECP) in adult patients (NCT04446182) as well as ibrutinib in patients over 12 years old (NCT02959944) as frontline cGvHD treatment.

For a risk-adapted, individualised approach to cGvHD management, not only the risk of relapse and infectious complications but additionally details of the pharmacological immunosuppression at onset of cGvHD may be considered (the intensity of any ongoing immunosuppression or time since termination of immunosuppression). Furthermore, the risk factors for cGvHD associated with poor prognosis (i.e., lung involvement, gastrointestinal involvement, hyperbilirubinaemia, thrombocytopenia and progressive onset) and the patient's general condition (Karnofsky/Lansky score) (91) may be of help to calibrate the intensity of first-line treatment.

Particularly for paediatric patients, the toxicity of long-term steroid therapy causes significant future problems (see Table 4) (114–138), such as effects on the musculoskeletal system resulting in growth and developmental retardation (117, 139). Therefore, the addition of an effective steroid-sparing agent and topical therapy is of crucial importance for long-term patient outcome.

**Table 4:** The main side effects of commonly used agents for cGvHD, other than infection risk.

**Therapy**	**Side effect**						
	**Blood**	**Cardiovascular**	**Visceral**	**Mobility**	**Neurological**	**Hormonal**	**Other**
Steroids (114–117)	Leucocytosis	Hypertension, metabolic syndrome, thrombosis	Peptic ulcer	Myopathy, avascular bone necrosis	Depression, behavioural changes (264), sexual dysfunction	Insulin resistance, hyperglycaemia	Striae, weight gain, hirsutism, glaucoma, cataract, fatigue
Mycophenolate mofetil (118–122)			GI toxicity, nausea diarrhoea, abdominal discomfort, hepatitis		Peripheral neuropathy		Increased risk of skin cancer, fatigue
Calcineurin Inhibitors (114)	Anaemia, thrombo-cytopenia	Hypertension, transplant-related microangiopathy	Acute and chronic nephropathy, tubular dysfunction (hyperkalaemia, hyponatraemia, hypomagnesaemia, hypercalciuria, and hyperuricaemia)	Peripheral neuropathy (264)	Central neuropathy, tremor, psychosis, PRES, seizures (264)	Impaired glucose tolerance, diabetes	Hirsutism, increased risk of skin cancer, fatigue
Sirolimus (114, 123)	Pancytopenia	Hypertension, hyperlipidaemia, peripheral oedema	Renal insufficiency, proteinuria, colitis, pancreatitis	Avascular bone necrosis			Pneumonitis, fatigue
Imatinib (124, 125)	Leukopenia	Peripheral oedema	Nausea Abdominal discomfort	Muscle spasms Stiffness	Sexual dysfunction		Oral ulcers, fatigue
Rituximab (111, 113, 125–128)	B-cell aplasia, hypo- or a-gammaglobulinaemia				Depression		Fatigue
Ibrutinib (129)	Low platelets, bleeding	Hypertension, cardiac arrhythmia	Nausea	Muscle spasms, peripheral neuropathy	Peripheral neuropathy		Oral ulcers (137, 138), fatigue
Ruxolitinib (130–132)	Pancytopenia, bleeding	Hypertension, hyperlipidaemia	Hepatitis, GI bleeding		Dizziness, headaches		Weight gain, fatigue
ECP (133–135)		Vascular access complications, thrombosis					

*This summary lists the most common or most severe persistent side effects of therapeutic regimens. For a full list of side effects for each agent, please refer to the most recent product information. cGvHD, chronic graft- vs.-host disease; ECP, extracorporeal photopheresis; GI, gastrointestinal; PRES, posterior reversible encephalopathy syndrome (114–122)*.

##### Topical Treatment and Ancillary Care

In general, topical treatment and ancillary care for cGvHD is less toxic than systemic therapy and can improve response, thereby facilitating systemic dose reduction with the aim to apply systemic immunosuppression at the lowest effective dose for the shortest possible duration. This approach allows for minimisation of treatment-related side effects, and, in case of high risk of relapse or infection, it may spare systemic immunosuppression saving the protective GvL effect. The latter aspect is supported by consensus opinion predominantly, and controlled data are scarce in this regard (140–142).

Ample topical treatment of cGvHD is important in mild cGvHD as systemic immunosuppression may not be required, while in moderate-to-severe cGvHD, topical treatment may hasten local responses in addition to systemic therapy. In patients with mixed responses (i.e., who have a response in one organ yet stable disease/progression in another organ) remaining symptoms may be addressed by topical treatment.

Of note, topical treatment in children bares two caveats: firstly, systemic levels of topical agents must be considered in infants because they have a larger surface area to body weight ratio than older patients and, secondly, the parents' assistance and compliance must be gained. In Table 5 (106, 135, 139–143, 158–167) we provide selected organ-specific modalities of topical treatment and ancillary care for use in daily clinical practice, providing paediatric data where possible. For more detail, we refer readers to comprehensive publications by Dignan et al., Wolff et al., and Carpenter et al. (108, 142, 143).

**Table 5:** Topical treatment and ancillary care for cGvHD.

**Organ**	**Topical therapy**	**References**	**Patients, ***N*** (age group)**	**Comments**	**Ancillary care**
Skin	Steroids	(108, 142, 143)	Review	Lichenoid and sclerodermoid cGvHD •Possible risk of cutaneous infection, skin atrophy and steroid acne •Interference with skin healing •Systemic side effects •Face: pimecrolimus preferred; if needed, low potency corticosteroids	•Emollients •Occlusive dressings •Systemic antihistamines •Exclusion of infection •Sun protection In erosions/ulcerations: •Microbiologic cultures •Topical antimicrobials, wound dressings •Consultation of wound care specialist and GvHD experienced dermatologist
	Narrowband UVB (311 nm)	(144)	10 (P/Ad)	Lichenoid and sclerodermoid cGvHD •Well-tolerated, feasible •3–5 times/week •Does not reach the dermal layers involved in deeper sclerotic cGvHD •Voriconazole and cotrimoxazole: increased phototoxicity •Possible risk of cutaneous neoplasm	
		(145)	3 (P/Ad)		
	PUVA bath	(146)	4 (P)	•Well-tolerated •3 times/week •Voriconazole and cotrimoxazole: increased phototoxicity •Possible risk of cutaneous neoplasm	
	UVA1	(147)	17 (P/A/Ad)	•Sclerodermoid cGvHD •Well-tolerated, feasible •3 times/week •Voriconazole and cotrimoxazole: increased phototoxicity	
		(148)	6 (P/A)		
	Pimecrolimus	(149)	1 (Ad)	Lichenoid and sclerodermoid cGvHD	
		(150)	1 (A)		
	Tacrolimus	(108, 142, 143)	Review	Lichenoid and sclerodermoid cGvHD •No skin atrophy •Possible systemic side effects in infants	
Mouth	Steroids	(151)	22 (P/Ad)	Caveat fungal overgrowth	•Topical analgesics •Therapy for oral dryness (e.g., salivary stimulants, sialogogues) •Routine dental care and prevention of related complications (i.e., dental decay) •Lips: topical tacrolimus or pimecrolimus preferred because of corticosteroid-associated atrophy of the lip vermillion
	Tacrolimus	(152)	22 (P/Ad)		
Eyes	Steroids	(153)	7 (P/A)	Caveat corneal thinning, infectious keratitis, glaucoma, cataract	•Exclusion of infection •Consultation of a paediatric and GvHD experienced ophthalmologist •Artificial tears, ocular ointments •Punctal occlusion, humidified environment, occlusive eye wear, moisture chamber eyeglasses, scleral contact lens
	Cyclosporine	(154)	Review	Burning sensation	
	Autologous serum eye drops	(143)		Well-tolerated	
Vulva and vagina	Steroids	(155)	33 (P/A)	Caveat fungal overgrowth	•Exclusion of coexisting infection •Water-based or silicone lubricants •Early gynaecology consultation •Avoid glycerin, paraben, fragrance and other additive products
	Oestrogen				
GI tract and liver	Steroids	(156)	15 (P/A)		•Exclusion of coexisting infection or gastroesophageal reflux •Avoidance of hepatotoxins •Dietary modification •Enzyme supplementation for pancreatic insufficiency •Gastroesophageal reflux management •Ursodeoxycholic acid
		(157)	33 (P/A)		
Lung	Steroids	(108, 142, 143)	Review		•Exclusion of coexisting infection •Fluticasone, azithromycin and montelukast (FAM) •To enhance mucociliary clearance: inhalation with hypertonic saline 3–6% •Optimal supportive care •Immunoglobulin substitution •Pulmonary rehabilitation
	Inhaled bronchodilators				

*A, adults; Ad, adolescents; cGvHD, chronic graft- vs.-host-disease; GI, gastrointestinal; P, paediatric; PUVA, Psoralen ultraviolet light A; UVB, ultraviolet light B*.

##### Steroid Refractoriness

Treatment of cGvHD aims to reduce symptoms, control activity of disease manifestations, improve OS and quality of life, and prevent impairment and tissue damage. Untreated cGvHD leads to disability and death. Steroids as first-line cGvHD therapy led to a complete response (CR) in 30–50% of patients, which may indicate that the remaining 50–70% have steroid-refractory or steroid-dependent disease. Therapy is usually long-lasting. The median duration of systemic cGvHD treatment was 28.7 months in one study of paediatric and adult patients (86). Among patients with cGvHD, approximately 50% discontinue systemic treatment within 7 years, 10% require continued systemic treatment beyond 7 years, and 40% experience recurrent malignancy or NRM (158).

In 2018, the following definitions of steroid-refractory and steroid-dependent cGvHD were suggested by the European Society of Blood and Marrow Transplantation (EBMT)-NIH-CIBMTR Task Force: (105).

- Steroid-refractory cGvHD (SR-GvHD): progression of cGvHD despite prednisolone ≥1 mg/kg/day for 1–2 weeks, or stable cGvHD without improvement for 1–2 months while on prednisolone ≥0.5 mg/kg/day
- Steroid-dependent cGvHD: two unsuccessful attempts, separated by at least 8 weeks in time, to taper steroids.

The incidence of SR-cGvHD is difficult to estimate. A prospective study by Martin et al. in adults (159) showed that >20% of the patients achieve CR or partial response (PR) to first line treatment based mostly on prednisone with or without calcineurin inhibitors with no secondary systemic treatment or recurrent malignancy at 1 year after the initial cGvHD treatment. This indicates a great need to search for and design new first-line treatment regimens.

#### Second- and Late-Line Therapy for cGvHD

So far there is no consensus regarding second and later lines of treatment for SR-cGvHD. There are numerous drugs and cellular therapy options that may be considered in this group of patients. Most of them were studied in retrospective analyses or small groups of patients, and there are very few prospective clinical trials regarding paediatric populations with cGvHD.

Paediatric data on the use of immunosuppressive and immunomodulating drugs in the treatment of cGvHD are summarised in Table 6 (62, 119, 120, 124, 126, 130, 133, 136, 160–205).

**Table 6:** Immunosuppressive and immunomodulatory agents used in the treatment of paediatric cGvHD.

**Therapeutic agent**	**Mechanism of action**	**Response**	**Comments**	
Mycophenolate mofetil (MMF)	Depletes guanosine nucleotides in T and B lymphocytes leading to inhibition of their proliferation (119)	ORR 60% in a study of 15 paediatric patients 3–16 years (220). Best responses in GI tract (60% CR), mouth (33% CR) and non-sclerodermatous skin involvement (43% CR). ORR 69% in a prospective study of imatinib + MMF for 13 paediatric patients (age 5–20 years) with sclerotic / fibrotic SR-cGvHD (160)	No benefit was found from adding MMF to first-line treatment for cGvHD (120)	
Rituximab	Humanised chimeric monoclonal anti-CD20 antibody that induces killing of CD20^+^ cells by direct and indirect mechanisms (126)	ORR 86.4% in 37 patients (age 8–57 years): 8/37 CR, 24/37 PR. The responses were better for skin, oral cavity and musculoskeletal involvement (161)	Attention must be paid to anti-infectious prophylaxis.	
Methotrexate	Multiple actions: (1) suppresses many inflammatory and immune reactions; (2) induces T-cell apoptosis; (3) increases the expression of long non-coding RNA p21, which regulates many immune and inflammatory processes; (4) modulates signalling pathways in T cells, macrophages, endothelial cells and fibroblast-like synoviocytes (162)	Meta-analysis by Nassar et al. (163) of 125 patients (age 2–60 years): ORR 77.6%, CR 49.6%, PR 28%. Best responses were achieved in skin (77%) and liver (72%); 2 out of 2 patients with pulmonary involvement responded.	Grade III–IV haematologic toxicities observed in 17.6%. Methotrexate is one of the most cost-effective drugs used in the treatment of SR-cGvHD (164)	
Tacrolimus	Calcineurin phosphatase inhibitor (inhibits T-lymphocyte signal transduction and IL-2 transcription) (165, 228).	ORR 46% in combination with MMF for refractory cGvHD in 26 patients (7 patients under 20 years old) (166)	79% treatment failure in 39 patients treated with tacrolimus after first-line treatment failure (CsA + prednisone) (167)	
Cyclophosphamide	Alkylating agent	ORR 53% in 13 patients (age 28–67) with SR-cGvHD (CR 1/13, PR 6/13) (168)	Very few retrospective studies. Three of three adults with cGvHD showed response in liver and oral cavity (169)	
mTOR inhibitor (sirolimus, everolimus)	Inhibits mTOR, a kinase regulating mRNA translation and protein synthesis; stops cytotoxic T-cell proliferation and dendritic cell activity; promotes generation of T_regs_; and has antifibrotic, antineoplastic and antiviral effects (170)	ORR 48.6% in 138 patients (7 patients under 20 years old) at 6 months when used with prednisone as frontline cGvHD therapy (171)	ORR 63–81% in SR-cGvHD in adult studies (172, 173) Main adverse events include renal toxicities (when used with CNIs), hyperlipidaemia, cytopenia and thrombotic microangiopathy.	
Pentostatin	Inhibitor of adenosine deaminase which is active mainly in lymphoid system cells, especially T cells.	ORR 53% in paediatric phase 2 trial of pentostatin for SR-cGvHD in 51 children, median age 9,8 years (175). ORR 55% in a prospective phase 2 trial (174) of 58 patients (age 5–64 years)—the response rate was better among patients <33 years old vs. >33 years old (77 vs. 37.5%).	Toxicity requiring drug discontinuation occurred in 25%. The drug had a significant steroid-sparing effect (175)	
Belumosudil	Selective Rho-associated coiled-coil–containing protein kinase 2 (ROCK2) inhibitor, decrease of IL-17 and IL-21	Best ORR 74–77% in 65 patients aged >12 years with persistent cGvHD after 2 to 5 prior systemic treatment lines (198)	Overall median time to response was 5 weeks (range, 4–66) 38% of subjects had ≥1 SAE; the most common was pneumonia (7%), nausea, diarrhoea, asthenia.	
Bortezomib	Reversible proteasome inhibitor. Inhibits T cells and prevents activation of dendritic cells that mediate antigen presentation and cytokine transcription	ORR 80% (10% CR, 70% PR) in 22 adults receiving bortezomib+prednisone for initial therapy (199) successful discontinuation of steroids in 2 of 3 paediatric patients with skin GVHD (200)	Main side effects: nausea, diarrhoea, thrombocytopenia, peripheral neuropathy	
Pomalidomide	Derivative of thalidomide (4,000-fold greater inhibition of TNF-α than thalidomide)	ORR 67% in 24 adults with SR-cGvHD at 6 months (201) ORR 54% in 13 adults with SR-cGvHD (only PR) (202)	Lack of paediatric data The most frequent adverse events: lymphopenia, infection, and fatigue, muscle cramps, tremors, neuropathy. May cause cutaneous inflammation early after HSCT	
Abatacept	Blocker of costimulatory signal—it binds to the costimulatory receptors CD80 and CD86 on antigen presenting cells and counteracts the costimulatory signal mediated by the ligand CD28 > T cell activation inhibitor	Best ORR 40% in a retrospective study of 15 adults (209) ORR 44% (PR) in a phase I study of 16 adults with SR-cGvHD (203)	Lack of paediatric data Serious infectious complications in 20% (mostly pulmonary)	
Tocilizumab	IL-6 receptor inhibitor	ORR 70% (PR) in a retrospective study of 11 adults with severe SR-cGvHD (204) $\frac{3}{4}$ paediatric patients with refractory cGvHD decreased NIH overall Grade by one (205)	Neutropenia, infectious complications	
Imatinib	Tyrosine kinase inhibitor; inhibits *BCR-ABL1* fusion protein and inhibits other tyrosine kinases of the PDGFR and TGF-β pathways which play a role in fibrosis.	ORR 79% (37/42% CR/PR) in refractory cGvHD with fibrotic features (19 patients, age 10–62 years) (124) ORR 76.9% in 13 paediatric patients with bronchiolitis obliterans (136 ) 36% PR (≥25% improvement) in range of motion of joints limited by skin fibrosis (20 patients, age 7-60 years) (62 ).	Oedema and fluid disturbances	
Ibrutinib	Tyrosine kinase inhibitor. Inhibits Bruton's tyrosine kinase which promotes B cell survival and IL-2-inducible T cell kinase which is involved in the selective activation of T cells.	ORR 85% (PR) at 6 months in 14 paediatric patients (median age 13,5 years) with cGvHD who completed the study (8/22 stopped ibrutinib by 3 months due to side effects or death) (129) ORR 41.1% at 48 weeks in a prospective study of 193 patients >12 years old in the first-line treatment (177)	FDA approval for adults with refractory cGvHD –ORR 67% in a study by Miklos et al. (176) High incidence of infections, bleeding disorders and hepatotoxicity. Paediatric pharmacokinetic studies are needed.	
Ruxolitinib	Selective JAK1/JAK2 inhibitor. JAK signalling plays a role in B-cell development and activation (178) and dendritic cell differentiation and migration (179). Ruxolitinib decreases T-cell proliferation and activation and reduces cytokine release (180). Data from murine models suggest that ruxolitinib does not inhibit GvL activity (181).	ORR 70–91%	High incidence of infection. Phase 3 REACH3 study: (197) significantly greater ORR compared to best available therapy (49.7 vs. 25.6%) at week 24. The most common adverse events were anaemia (29.1%), thrombocytopenia (21.2%), hypertension (15.8%), and pyrexia (15.8%).	
**Ruxolitinib for cGvHD in paediatric patients**				
**References**	***N***	**Age range (years)**	**Response**	
Mozo et al. (182)	19	2–16	ORR 91%, CR 8.3%	
Yang et al. (183)	36	3–17	ORR 80.6%, CR 27.7%	
Wang et al. (130)	20	5–26	ORR 70%, CR 10%	
Moiseev et al. (184)	17	2–17	ORR 81%, CR 20%	
Uygun et al. (185)	29	0.3–17	ORR 80%	
Gonzalez Vicent et al. (186)	9	0.5–18	ORR 89%	
Escamilla Gomez et al. (196)	56 (7 patients <14 years old)	0–73	Best ORR 57,1%	
Zeiser et al. (197)	330	12+	REACH 3—Phase III randomised study (NCT03112603) Best ORR 49,7%	
**ECP for the second-line treatment of cGvHD** **References**	***N***	**Age range (yrs)**	**Corticoid sparing**	**ORR (%)**
Salvaneschi et al. (187)	14	5.4–18.1	Yes	64
Seaton et al. (188)	28	18–51	No	36
Couriel et al. (189)	71	5–70	Yes	61
Kanold et al. (133)	27	5–18	No	73
Perseghin et al. (190)	12	9–17	NA	80
Dignan et al. (191)	82	14.1–69.5	Yes	79
Hautmann et al. (192)	32	6–67	No	44
Berger et al. (193)	10	7–18.5	Yes	40
Perotti et al. (194)	23	Mean 11.8	Yes	69.5
Messina et al. (195)	44	0.3–20.5	Yes	73

*ALL, acute lymphoblastic leukaemia; cGvHD, chronic graft- vs.-host disease; CML, chronic myeloid leukaemia; CNI, calcineurin inhibitor; CR, complete response; CsA, cyclosporine A; ECP, extracorporeal photopheresis; GI, gastrointestinal; JAK, Janus kinase; MMF, mycophenolate mofetil; mTOR, mammalian target of rapamycin; N, number of patients; ORR, overall response rate; PDGFR, platelet-derived growth factor receptor; PR, partial response; SR, steroid refractory; TGFβ, tumour growth factor β*.

Second-line therapy should include agents with high efficacy and a good safety profile. In ALL patients, it is also important to spare the GvL effect. It is known that ECP preserves the antiviral and anti-leukaemic effect (206) and has a very low incidence of side effects. TKIs enhance the anti-leukaemic effect and are highly effective in SR-cGvHD but some studies reported a high incidence of infectious complications. Anyway, classical immunosuppressive agents like high-dose steroids, mycophenolate mofetil, rituximab, methotrexate, cyclophosphamide, pentostatin and mTOR inhibitors still find their place in SR-cGvHD management. Some of therapies are more effective than others for specific cGvHD manifestations, which also should be taken into account when selecting second and later lines of therapy (Table 6).

##### New and Emerging Therapies

In recent years various novel agents have been tested in the treatment of cGvHD. Among them tyrosine kinase inhibitors (TKIs) found their place in the therapy of SR-cGvHD and were approved by FDA in this indication. We discuss them below.

Belumosudil, a selective ROCK2 inhibitor, has been shown to be effective in recipients over 12 years of age with persistent cGvHD who failed 2–5 prior systemic lines of treatment and was approved by FDA in this age group (198). It decreases production of IL-17 and IL-21, which are pro-inflammatory cytokines and mediators of autoimmune disorders like rheumatoid arthritis and systemic lupus erythematosus. In a phase II clinical trial of 65 participants with predominantly severe cGvHD complete resolution was observed in 6% of patients and partial response in 69%, with a duration of response for a median of 50 weeks.

Bortezomib is a reversible proteasome inhibitor and has an inhibitory effect on B cells and plasma cells (207). It showed efficacy in murine models of cGvHD with maintained graft vs. tumour effect (208). Its efficacy in the initial therapy of cGvHD (together with prednisone) was evaluated in a study of 22 adults and showed 80% ORR (199). Paediatric data on its use in cGvHD treatment are very scarce.

Pomalidomide is a thalidomide derivative with 4,000-fold greater inhibition of TNF-alpha, which was originally used in the treatment of multiple myeloma. It has been evaluated in several adult studies for the treatment of SR-cGvHD with 54–67% ORR observed (201, 202). Paediatric data are lacking.

Abatacept, a costimulatory signal blocker which inhibits T cell activation, has been also evaluated in small cohorts of adult patients with SR-cGvHD and showed 40–44% ORR. As for pomalidomide, paediatric data are missing (203, 209).

Tocilizumab, a humanised IgG1 interleukin 6 (IL6)-receptor antibody, has shown efficacy in aGvHD and cGvHD. IL6 plays a significant role in the initiation of the inflammatory response, leads to increased immunoglobulin production by B cells and decreased differentiation of T_regs_ (210). It was investigated in a retrospective adult study showing ORR of 70%, as well as in a retrospective paediatric case series (204, 205). Infections were the primary adverse events associated with tocilizumab administration.

##### Tyrosine Kinase Inhibitors

TKIs are considered promising drugs in the treatment of SR-cGvHD. Tyrosine kinases play a role in cell processes such as differentiation, proliferation, anti-apoptosis, and B- and T-cell signalling. TKIs have the potency to block B- and T-cell activation and to inhibit the transcription of genes encoding pro-inflammatory cytokines (211). They have been used in the treatment of haematological malignancies including acute leukaemia, B-cell lymphoma, chronic lymphocytic leukaemia and chronic myeloid leukaemia (CML). Their inhibitory effect on B and T cells led to their use in preclinical and clinical trials of cGvHD treatment. The use of imatinib, ibrutinib and ruxolitinib for cGvHD treatment in paediatric patients is summarised in Table 6.

In a mouse model of cGvHD, it was shown that animals lacking BTK in B cells or IL-2-inducible kinase in T cells did not develop cGvHD. In addition, activation of T and B cells from patients with active cGvHD was inhibited by ibrutinib blockade of BTK and IL-2-inducible kinase. Based on these pre-clinical data, the first clinical trials with ibrutinib in cGvHD were designed (212). In 2017, ibrutinib became the only drug approved by US Food and Drug Administration (FDA) for the treatment of SR-cGvHD in adults; this approval was based of the study data by Miklos et al. (176). There are ongoing clinical trials on the use of ibrutinib for cGvHD, including in paediatric patients (NCT02959944).

Ruxolitinib was approved by the FDA in 2019 for salvage therapy in patients with aGvHD. Several retrospective studies have evaluated ruxolitinib in the treatment of SR-cGvHD in adults, with a 85.4% ORR observed in one multicentre retrospective survey (131). There was also a low recurrence rate of the underlying malignancy. Ruxolitinib has been evaluated also in paediatric patients with cGvHD, with a 70–91% ORR observed (see Table 6). The favourable results of the phase 3, randomised, multicentre study REACH 3, which investigated the efficacy of ruxolitinib in SR-cGvHD patients ≥12 years of age as add-on therapy to steroids and in comparison to best available therapy, formed the basis for the FDA approval of ruxolitinib in September 2021. Prospective clinical trials and pharmacokinetic studies of ruxolitinib in paediatric patients are currently ongoing [REACH 4 in aGvHD and REACH 5 in cGvHD (132)].

#### Immunomodulatory Interventions

##### Extracorporeal Photopheresis

**Figure 1:**
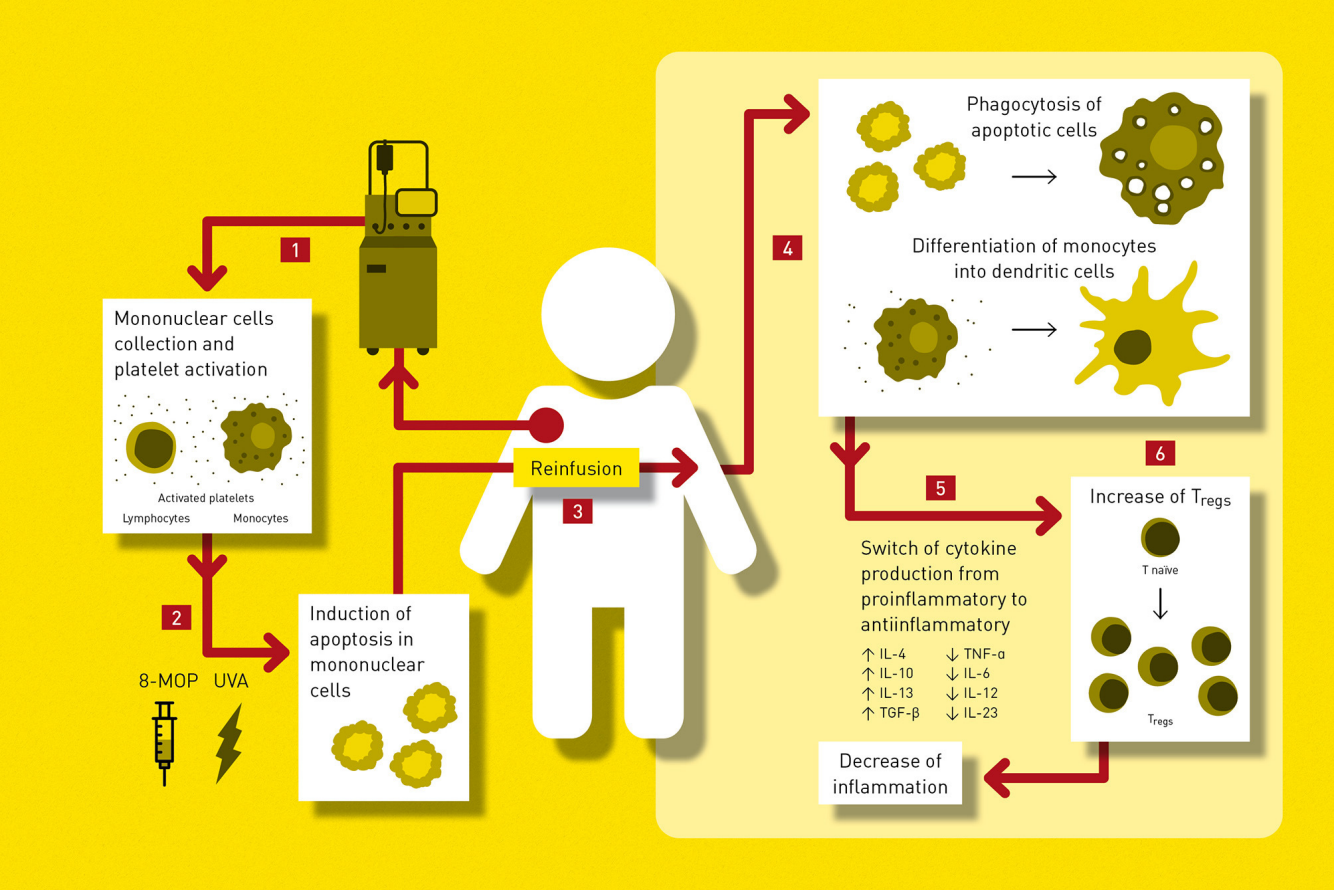
Proposed procedure of ECP and its hypothesised mechanism of action. 1. Collection of mononuclear cells (MNC) during leukapheresis from the peripheral blood and activation of platelets by the plastic surfaces of the tubing system. 2. *Ex vivo* incubation of leukapheretic product with a photosensitizing agent 8-methoxypsoralen (8-MOP) followed by ultraviolet-A light (UVA) irradiation which initiates apoptosis in MNC including lymphocytes. 3. Reinfusion of the ECP product. 4. Process of apoptosis continues in ECP exposed cells for days resulting to phagocytosis by antigen presenting cells (APC). Activated platelets engage with monocytes promoting their differentiation into dendritic cells (DC). 5. The internalisation of apoptotic cells decrease the inflammatory reaction of phagocytes, induces antigen specific immunotolerance and lower production of proinflamatory cytokines while increasing antiinflamatory cytokines production (213, 214). ECP- induced DC initiate T-cell tolerance with an increase of Th2 cytokines including IL-4, IL-10, IL-13 and TGF-β, while production of Th1 cytokines is suppressed (215). 6. APC promote generation of regulatory T-cells (T_regs_) (216). MNC, mononuclear cells; 8-MOP, 8-methoxypsoralen; UVA, ultraviolet A light; APC, antigen presenting cells; DC, dendritic cells; T_regs_, regulatory T-cells.

ECP is an immunotherapy using the recipient's leukocytes to modulate inflammatory immune dysregulation in persons with cGvHD (213). The main technique fundaments of ECP are comprehensively outlined in Figure 1. This technique was approved by both the FDA and European Medicines Agency (EMA) for T-cell cutaneous lymphoma treatment (216). In the post HSCT setting, ECP can be applied both for the treatment of acute and chronic SR-GvHD (217).

The exact working mechanisms of ECP are incompletely understood but its effects might be considered on different levels, as outlined below.

Firstly, ECP might have a mechanical effect (irrespective of the disease for which it is applied) driven by the movement of blood through plastic tubing. Changes in monocyte and dendritic cell differentiation and maturation have been documented when blood is processed over plastic, probably *via* activated platelet signalling (213). Additionally, 8-MOP and exposure to UVA induces cross-linking damage to DNA in leukocytes, which induces apoptosis. The uptake of apoptotic cells by activated dendritic cells leads to changes in dendritic cells and a switch to a more tolerogenic phenotype (217, 218). This change in dendritic cell morphology and function has been demonstrated in several different diseases and likely represents the primary effect of ECP (213, 214, 217).

Other effects of ECP occur downstream and reflect the disease process that is being treated, the age of the patient and extent of organ damage. Importantly, ECP can induce changes not only in cells in the inoculum which are directly exposed to 8-MOP and UVA but also in cells that are not directly harvested, suggesting that the immunomodulatory effects of ECP propagate beyond directly treated cells. ECP has been shown to induce a switch from a Th1- to Th2-type response with immunomodulatory cytokines in GvHD (213, 215). A switch from proinflammatory to anti-inflammatory cytokine production (with a decrease in IFN-γ, TNF-α, and IL-2 secretion and an increase in TGF-β serum levels) as well as increase in T_reg_ numbers has been described (214, 216). Additionally, some authors have postulated that ECP impacts on B-lymphocyte homeostasis, with a decrease in CD19^+^/CD21^−^ B-lymphocyte subsets, where others have described the possible expansion of CD8^+^ memory cells and differentiation of monocytes to immature antigen-presenting cells (219, 220). Therefore, the immune modulatory effect of ECP appears to be a complex response to the whole procedure, as depicted in Figure 1.

In contrast to conventional immunosuppression, ECP is safe and has limited side effects, confined mainly to risks associated with use of an indwelling central venous catheter (including infection), hypotension and photosensitivity related to 8-MOP exposure (215). In small children, the leukapheresis procedure itself may be technically challenging (215, 221).

**Figure 2:**
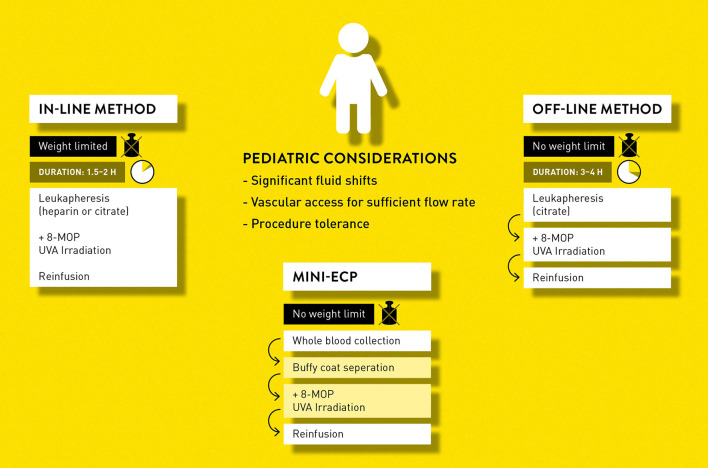
Different approaches to extracorporeal photopheresis.

Currently, there are three techniques in use for ECP: the in-line method (“closed” system), the off-line method (“open” system) and so-called mini-ECP which we briefly describe in Figure 2.

Importantly, ECP is not associated with an increased risk of infectious complications, likely because it spares antigen-specific activity against novel and recall antigens. Further benefits are the potential preservation of the GvL effect and—in contrast to systemic immunosuppressive treatment—the absence of metabolic or toxic side effects (222, 223).

Abu-Dalle et al. published a systematic review of the literature in 2014 including 9 studies (1 randomised trial) of ECP for cGvHD in 323 patients aged 1.4–67 years. In a pooled analysis, the ORR for cGvHD overall was 64% (95% confidence interval [CI], 47–79%) and the proportion of patients with CR in various organs was 26% (95% CI, 5–55%). The ORR for skin manifestations was 71% (95% CI, 57–84%), for gut it was 62% (95% CI, 21–94%), for liver it was 58% (95% CI, 27–86%), for oral mucosa it was 63% (95% CI, 43–81%), for the musculoskeletal system it was 45% (95% CI, 18–74%) and for the lung it was 15% (95% CI, 0–50%) (224). The majority of reported paediatric data are predominantly derived from non-randomised, single-centre or retrospective studies and are summarised in Table 6. Treatment schedules and durations of ECP for paediatric cGvHD management vary but most often involve two procedures applied every other week. The optimal approach has not been established yet.

The benefits of ECP include reduction in the need for conventional immune suppression, with corresponding reduction in the risk of infection, secondary malignancies and adverse effects attributable to those conventional immunosuppressive therapies. For example, patients reducing or ceasing glucocorticoids may have normalisation of blood pressure and blood glucose. Based on its efficacy and the excellent safety profile, several expert groups have reached the consensus that ECP has an established place as second-line or adjuvant therapy in cGvHD (216).

In 2013–2014, the Paediatric Diseases Working Party (PDWP) of the EBMT conducted a survey on the use of ECP in paediatric GvHD treatment in routine clinical practice; 52 EBMT centres responded (19%). Results of the analysis revealed that the majority of centres used ECP as an “add on” treatment during various lines of GvHD therapy in patients with a high risk of relapse or infection (81%) or with comorbidities (88%). Of note, 85% of responding centres agreed that, in children, a non-malignant disease and no need of GvL may be an indication for early implementation of ECP within a multimodal GvHD treatment schedule (Lawitschka et al., unpublished results).

The NIH 2020 initiative set the stage for future GvHD research projects including the further evaluation of ECP within a pre-emptive therapeutic setting for well-defined forms of highly morbid cGvHD, since ECP does not increase risk of relapse or infection. The expert group recommended the evaluation of ECP as a first-line therapeutic agent, applying rigorous biomarker panels pre- and post-intervention. Databases including biobanks should be analysed for a predictive biomarker of response to ECP (12, 13, 225, 226).

##### Mesenchymal Stromal Cells

Mesenchymal stromal cells (MSCs) are a heterogeneous precursor cell population with some degree of pluripotency. Potential usefulness for treatment of GvHD was suggested early on as MSCs can modulate immune responses (227). Tissue regeneration properties were also noted.

According to current hypotheses, MSCs are injected as a “pro-drug.” They do not begin to secrete relevant mediators until they are immersed in an environment with certain cytokines, specifically IFNγ (228, 229), which is not a dominant mediator in cGvHD (230).

Clinical outcomes of studies provide conclusions that are limited only for the specific MSC product applied and clinical situation for which they were studied. In meta-analysis by Tarifa et al. infusion of MSCs (of variable provenance, variable dose, schedule, etc.) was associated with reduced cGvHD incidence (relative risk, 0.64; 95% CI, 0.47–0.88; *I*^2^ = 0%) and a trend toward lower incidence of extensive cGvHD (relative risk, 0.50; 95% CI, 0.25–0.10; *p* = 0.05), both in adults and children (231). Fisher et al. came to essentially similar conclusions (232) as does the meta-analysis by Wang et al. (233), albeit restricted to children.

Outcomes were reported in several case series comprising fewer than 100 patients and MSCs of various provenance and dose. While they may appear overall satisfactory, it is very important to bear in mind that all of these studies lack control groups, which could have answered the question of attributability of the improvement to the MSC infusion, i.e., could have distinguished between “improvement” and “response.”

Alternatively, proving the hypothesis that prophylactic infusion of MSCs might be able to prevent cGvHD is hampered by the poor predictability of (severe) cGvHD and its relatively low prevalence. Work by Lazarus et al., reports a high frequency of cGvHD (of 61% in patients surviving to day 90, almost a quarter of whom had severe cGvHD) which does not suggest a prophylactic benefit (234). In that study, MSCs were co-administered with the graft. A later double-blinded trial of umbilical cord blood MSCs (235) investigated this issue further. In a 1:1 randomised assignment, 124 haploidentical transplanted patients received umbilical cord blood MSCs or control (saline). Although the treatment schedule itself is described in somewhat vague terms, a signal indicating efficacy is reported. Whether dose, schedule (especially timing relative to the transplantation), source of MSCs or any other quality attribute of the MSCs is responsible remains elusive. The promising data certainly encourage further exploration of the issue.

To summarise, the role of MSCs in cGvHD treatment is unclear. For a specific preparation of umbilical cord blood MSC efficacy was demonstrated in a prophylactic setting in haploidentical transplantation, which begs confirmation.

#### Real-World Response Evaluation

The appropriate assessment of cGvHD treatment response is essential for making optimal therapeutic decisions and, thus, for optimising final outcomes of cGvHD treatment. The 2014 NIH consensus criteria on diagnosis and grading include definitions of overall and organ-specific therapeutic response in cGvHD for use in clinical trials (135).

The NIH consensus project recommends that clinicians assess organ-specific response for the skin, mouth, liver, upper and lower gastrointestinal tract, oesophagus, lung, eye, and joint/fascia (236).

Three general categories of overall response are proposed:

- CR: resolution of all manifestations in each organ
- PR: improvement in at least 1 organ or site without progression in any other organ
- lack of response: unchanged, mixed response, or progression

Regarding timepoints for assessment, response should not be assessed earlier than 8 weeks after induction of treatment. Subsequent measurements should be made at regular intervals, for example every 3 months, and whenever a new systemic immunosuppressive treatment is started or the patient stops treatment (1, 236). Generally, a measure of success in cGvHD treatment is the complete discontinuation of therapy or complete disease control on unimodal immunosuppressive treatment at a low dose.

#### Tapering Systemic Immunosuppressive Treatment

There is no “gold standard” for tapering schedules of cGvHD treatment because randomised prospective trials are lacking. Therefore, expert-based recommendations compensate for the lack of evidence-based data.

The choice to taper treatment should be patient specific and may start with the agent that is either less well-tolerated by the patient or that has more toxic side effects. The schedule of taper may best be guided by the organ pattern and severity of cGvHD as well as the patient's individual risk of poor outcomes of cGvHD, concomitant comorbidities and infectious complications. In paediatric patients, tapering should usually start with steroids because of the broad spectrum of possible adverse effects with these agents and to allow for best possible growth and development of the child.

Generally, drugs should be withdrawn gradually, one at a time, after gaining an objective clinical response to therapy. In our opinion, regular clinical examinations in shorter intervals, such as once or twice weekly in moderate-to-severe cGvHD, are important. Discussions and shared management decisions within a multidisciplinary team are strongly recommended. We have summarised different published approaches to tapering in Table 7 (106, 139, 141, 237).

**Table 7:** Summary of recommended approaches for the taper of immunosuppressive agents used in the treatment of cGvHD (review of recent literature).

**References**	**Timing of taper initiation**	**Approach to taper**	**Approach to dose increase in the event of cGvHD relapse or exacerbation**
Sarantopoulos et al. (141)	After 3–4 weeks of the initial prednisone dose.	Not specified	Not specified
Wolff et al. (139)	As soon as disease control has been achieved.	Not specified	If cGvHD flares during steroid taper, increasing the dose by 1 or 2 taper steps may be enough to control symptoms.
Jacobsohn (106)	After 2 weeks of the initial prednisone dose.	Taper to alternate-day prednisone by 1–2 months.	Not specified
Flowers and Martin (237)	As soon as clinical improvement is achieved.	20–30% dose reduction every 2 weeks, with smaller absolute decrements toward the end of the taper schedule; the prednisone dose is reduced to 0.1 mg/kg every other day within 22 weeks; it equates to adrenal replacement therapy and is continued for at least 4 weeks.	2-log increase in dose with daily administration for 2–4 weeks, followed by resumption of alternate-day administration which is continued for at least 3 months before next attempt of taper.

*cGvHD, chronic graft vs. host disease*.

If cGvHD exacerbation occurs during the taper, other contributing causes, especially infections, must be excluded followed by a swift dose escalation. In the event of unresponsiveness or progression of cGvHD after 4 weeks, a new agent should be introduced. The same applies after two unsuccessful attempts to taper therapy. Ineffective treatment should be tapered and discontinued after successful induction of the new treatment to avoid unnecessary immunosuppression.

However, management of paediatric cGvHD requires continuous recalibration of immunosuppressive treatment in order to avoid over- or undertreatment. Usually, in paediatric patients the treatment intensity decreases over time and a specific threshold can be set individually for each patient by repetitive attempts to decrease treatment intensity.

#### Anti-infectious Prophylaxis

It is important to recognise that the complete management of cGvHD includes optimal supportive care. During cGvHD, patients are immunocompromised due to both immunosuppressive medication and immune dysregulation by cGvHD itself. The prolonged use of immunosuppressants in cGvHD is common (with only 18% of patients being off immunosuppressive therapy after 2 years in a combined paediatric/adult study) and is associated with an increased incidence of infection and mortality (238). cGvHD is a risk factor for bacterial, fungal and viral infections (239–241) and increased TRM (240, 242).

Therefore, prophylaxis against multiple types of infection is indispensable to minimise the risk of life-threatening infections (243). The 2014 NIH consensus project included recommendations on ancillary therapy and supportive care in cGvHD, including the strength of each recommendation (143). Recently, paediatric expert recommendations stemming from workshops of the EBMT PDWP were published regarding the prevention of infections in patients after HSCT (244).

##### Antibacterial Prophylaxis

In patients with cGvHD, the risk of infections caused by encapsulated bacteria is more than double that in those without cGvHD (245).

Prolonged antibiotic prophylaxis is recommended only for preventing infection with *S. pneumoniae* among cGvHD patients receiving active cGvHD treatment (level A-III) (243, 245, 246). Oral phenoxymethylpenicillin has been shown to prevent encapsulated bacterial infection and, thus, may be suitable (level A-III) (243, 245). However, it is recommended to make a choice of antibiotic agent according to local antibiotic susceptibility data (243, 246).

##### Pneumocystis Jirovecii Prophylaxis

In general, patients with active cGvHD taking immunosuppressive treatment (especially multimodal treatment including steroids) and/or with neutropenia and/or with CD4^+^ T cells <200 × 10^9^/L may be at risk of *Pneumocystis jirovecii* infection, taking into account that the initially HIV-derived CD4^+^ T-cell threshold has been not evaluated in the cGvHD setting and *Pneumocystis jirovecii* infections have been observed in patients above the proposed threshold. For prophylaxis against *Pneumocystis jiroveci* interstitial pneumonia, trimethoprim/sulfamethoxazole is recommended (level A-I) (7).

##### Antifungal Prophylaxis (Systemic and Topical)

If tolerated, a mould-active azole is recommended for prophylaxis in patients undergoing treatment for cGvHD (level A-I) (7, 243). Suitable agents include posaconazole and voriconazole (level A-I) or itraconazole with regular monitoring of plasma levels (level B-II) (243). If there is a history of invasive aspergillosis, secondary prophylaxis using antimycotics that are active against *Aspergillus* (level B-I) including weekly or biweekly liposomal amphotericin B should be administered (7, 247).

##### Antiviral Prophylaxis

In at-risk patients, the stringent monitoring of cytomegalovirus (CMV) levels by quantitative polymerase chain reaction (qPCR) should be continued throughout the period of cGvHD (level B-I) to enable pre-emptive treatment or maintenance of prophylactic management if needed (89, 243). Due to the high risk of post-transplant lymphoproliferative disease, it also is reasonable to monitor patients with cGvHD on T cell suppressive agents (i.e., a CNI, mycophenolate mofetil, or ruxolitinib) for Epstein-Barr virus reactivation by qPCR (243, 248).

In patients who are seropositive for herpes simplex virus or varicella zoster virus, acyclovir is recommended to prevent reactivation (level B-II) (7).

##### Toxoplasmosis Prophylaxis

In patients who were seropositive for toxoplasma pre transplant, there is a risk of reactivation during cGvHD treatment. Regular monitoring by qPCR is recommended. Of note, *Pneumocystis jirovecii* prophylaxis with trimethoprim/sulfamethoxazole potentially may be protective against toxoplasmosis because the majority of post-transplant cases occur in patients not receiving this prophylactic medication (243).

##### Tuberculosis Prophylaxis

If there is a history of tuberculosis, secondary prophylaxis using isoniazid should be used (level C-III) (7).

##### Intravenous Immunoglobulin

Substitution of polyvalent immunoglobulins either intravenously or subcutaneous is recommended in the presence of IgG deficiency (below 400 mg/dL) post transplant, post rituximab treatment and in patients with recurrent infections (7, 244). Immunoglobulin substitution does not inhibit the immune response to inactivated vaccines. For live virus vaccines, vaccination should be delayed until the patient is immunocompetent (at least 24 months post HSCT).

##### Vaccination of Patients and Close Contacts

There are data on vaccination responses in children after HSCT but very limited data are available specifically in children with cGvHD. A prospective study by Meisel et al. reported on the safety and immunogenicity of a heptavalent pneumococcal conjugate vaccine (7vPCV) administered to 53 children. Patients were immunised with 3 consecutive doses (at monthly intervals) starting 6–9 months after HSCT (249). Ten of the 53 patients had been on systemic immunosuppressive treatment, while patients with uncontrolled cGvHD were excluded. There were indications that the responses to 7vPCV in patients with active cGvHD were suboptimal, with low B cells and low IgG being risk factors for a suboptimal response (249, 250). Data from a combined paediatric/adult cohort where pneumococcal conjugate vaccination was triggered by milestones in immunity (CD4^+^ cells >200 × 10^6^/L and IgG >0.5 g/L), show that cGvHD patients respond just as well as patients without cGvHD to vaccination but are vaccinated significantly later after transplant than patients without cGvHD (the median time 1.8 vs. 1.1 year post HSCT, respectively) (251).

Importantly, there is no evidence that inactivated vaccines induce or aggravate GvHD (252, 253) and, therefore, the start of vaccination (or revaccination) with a diphtheria, tetanus, acellular pertussis, polio, hepatitis B and *Haemophilus influenzae* type B combination vaccine (DTaP/IPV/HBV/Hib) and 13-valent pneumococcal conjugate (PCV13) vaccine is recommended 6 months after allogeneic HSCT for patients with and without cGvHD (7, 244, 254). Cordonnier et al. showed that a fourth dose of PCV13 increased antibody levels significantly in children and this has been implemented in the current EBMT recommendations (level A-ll) (169, 255). The additional effectiveness of the polysaccharide vaccine Pneumo23 is potentially limited in patients who suffer from cGVHD after HSCT (244, 255).

In view of the especially high risk of encapsulated bacterial infection in cGvHD, all patients with cGvHD should receive vaccination against *Haemophilus influenzae* (level B-1) and *Streptococcus pneumoniae* (level A-ll) (243, 249, 254). Conjugate vaccines, which also achieve good vaccination success in infants, are preferred (7, 254).

Serum tests are recommended to monitor response to vaccination in patients receiving immunosuppression to assess the immunologic response to vaccination and/or need for subsequent booster immunisation (7, 244, 254).

Recommendations for optional and conditional vaccines can be found in the EBMT recommendations by Ifversen et al. (244). Of the recommended inactivated vaccines, influenza vaccine can be given from 4 to 6 months post transplant and immunisation should be repeated on an annual basis (7, 243, 244, 253). However, it has been observed that a greater percentage of adults with cGvHD do not respond to the H1N1 vaccine in comparison to healthy individuals (256). This is of particular interest in the light of the coronavirus pandemic and mass vaccination with COVID-19 vaccine of all patients with ALL, where impaired responses have indeed also been observed to the COVID-19 vaccine. The recommendations are continuously updated but the EMA currently advises to give three doses of COVID-19 vaccine to all adult immunocompromised patients (recommendations published on 04/10/2021, https://www.ema.europa.eu/en/news/comirnaty-spikevax-ema-recommendations-extra-doses-boosters) (257). As the vaccine has recently been EMA- approved for use in children over 5 years old it is likely that this recommendation will soon include children with ALL of 5 years and older, and after transplant regardless of cGvHD development (258). There are strong indications that patients with B cell depleting therapies impairing their antibody responses, are still able to mount adequate T cell responses against natural infection and COVID-19 vaccination (259, 260).

A strong recommendation is that live vaccines must not be administered in patients with cGvHD (level A-I) (243, 244, 254).

Household contacts should also receive routine vaccinations plus the seasonal influenza vaccine (254) and the COVID-19 vaccine (see EBMT website, COVID-19 vaccines).

## Personalised Management of Paediatric cGvHD

The 2020 NIH initiative clearly set out all the unmet needs in paediatric cGvHD management and pointed out that future efforts must aim for prompt recognition and intervention to limit organ damage and significant morbidity (11, 12).

Nonetheless, despite the advances brought through and driven by the 2014 NIH consensus conference, the diagnosis of paediatric cGvHD remains challenging in daily clinical practice since clinical onsets can be infection associated and insidious. Moreover, patients may present with clinical manifestations of cGvHD beyond the NIH-defined diagnostic and distinctive features. These are referred to as “associated cGvHD symptoms” and may consist of endothelial dysfunction and polyserositis, immune-mediated cytopenias, and atypical manifestations regarding the kidneys, the central and peripheral nervous system and others (226) (see supplemental cGvHD documentation form in Supplementary Material). However, standardised diagnostic criteria for associated manifestations are lacking and may be missed as being cGvHD associated. These atypical cGvHD manifestations are understudied in paediatric patients but may contribute significantly to morbidity and mortality and may share cGvHD pathophysiology (67). An additional challenge can be the differentiation of cGvHD manifestations from pre-existing toxicities and specific residual phenotypes of inborn errors appearing alongside paediatric cGvHD symptoms.

Another problem specific to cGvHD is the waxing and waning nature of the disease with high inter- and intraindividual heterogeneity. This impedes clinicians' decisions on when and how to best implement therapeutic agents, with the added difficulty of a lack of standardised recommendations, including taper schedules for treatments.

A further difficulty lies in how and when to best implement therapeutic approaches for the individual patient. Many research activities have provided new pathophysiological insights allowing for therapeutic approaches that may more accurately target involved pathways. However, substantially fewer data are available on how the various pathways intersect and how they apply to the various phenotypes of cGvHD. Of note, single-target inhibitors may have a beneficial or detrimental effect at different phases of immune cell development and immune dysfunction. In this regard the results of a randomised phase 2 trial evaluating the response of pomalidomide in 34 adult patients with moderate to severe cGvHD may serve as an example: authors reported that the use of pomalidomide early after HSCT may cause cutaneous inflammation in contrast to the treatment responses observed in late sclerotic cGvHD (201).

Another difficulty is that the rather promising results from early studies of these agents are yet to be confirmed in large prospective studies, and our understanding of drug interactions in children is currently incomplete. The FDA approval of ruxolitinib as the first agent for SR-cGvHD in patients over the age of 12 years in September 2021 will likely change the cGvHD field, but paediatric data from large prospective trials are missing. There is an ongoing REACH 5 trial evaluating ruxolitinib in patients under the age of 18 years with moderate to severe cGvHD.

Moreover, with respect to difficulties in the clinical management of paediatric cGvHD, a plethora of potential infections and drug-induced toxicities make a patient-specific approach of crucial importance. In this regard, the 2020 NIH initiative has emphasised the benefit of applying immunomodulatory agents as opposed to broad immunosuppressive agents (225).

A comprehensive review on the management of cGvHD in children was provided by Jacobsohn (106), but since that publication major advances, as outlined in detail within this manuscript, have been made and an update is pending. Recently, an individualised and patient-centred cGVHD management offering continuing care embedded in a multidisciplinary team has been described (103, 141). To fill this gap regarding paediatric cGVHD patients was the central aim of this manuscript.

In consideration of the unmet needs as outlined above, coupled with the debilitating morbidity of the disease, we have developed a model for a personalised approach for the management of paediatric cGvHD. This model integrates published evidence, expert opinions, clinicians' experience and patient-specific considerations.

### Holistic View of Paediatric cGvHD and Associated Manifestations (The See-Saw of cGvHD)

We propose that clinicians take a holistic view of paediatric cGVHD interpreting classical cGvHD, atypical cGvHD and other manifestations not only in the context of allo/auto-immunity after HSCT but rather as a kind of chronic graft dysfunction. This chronic graft dysfunction of the transplanted immune system involves multiple layers and effectors of the innate and adaptive aberrant immune system (30) which interfere with functional tolerance; chronic inflammation mediated by GvHD and/or infections play a central role.

**Figure 3:**
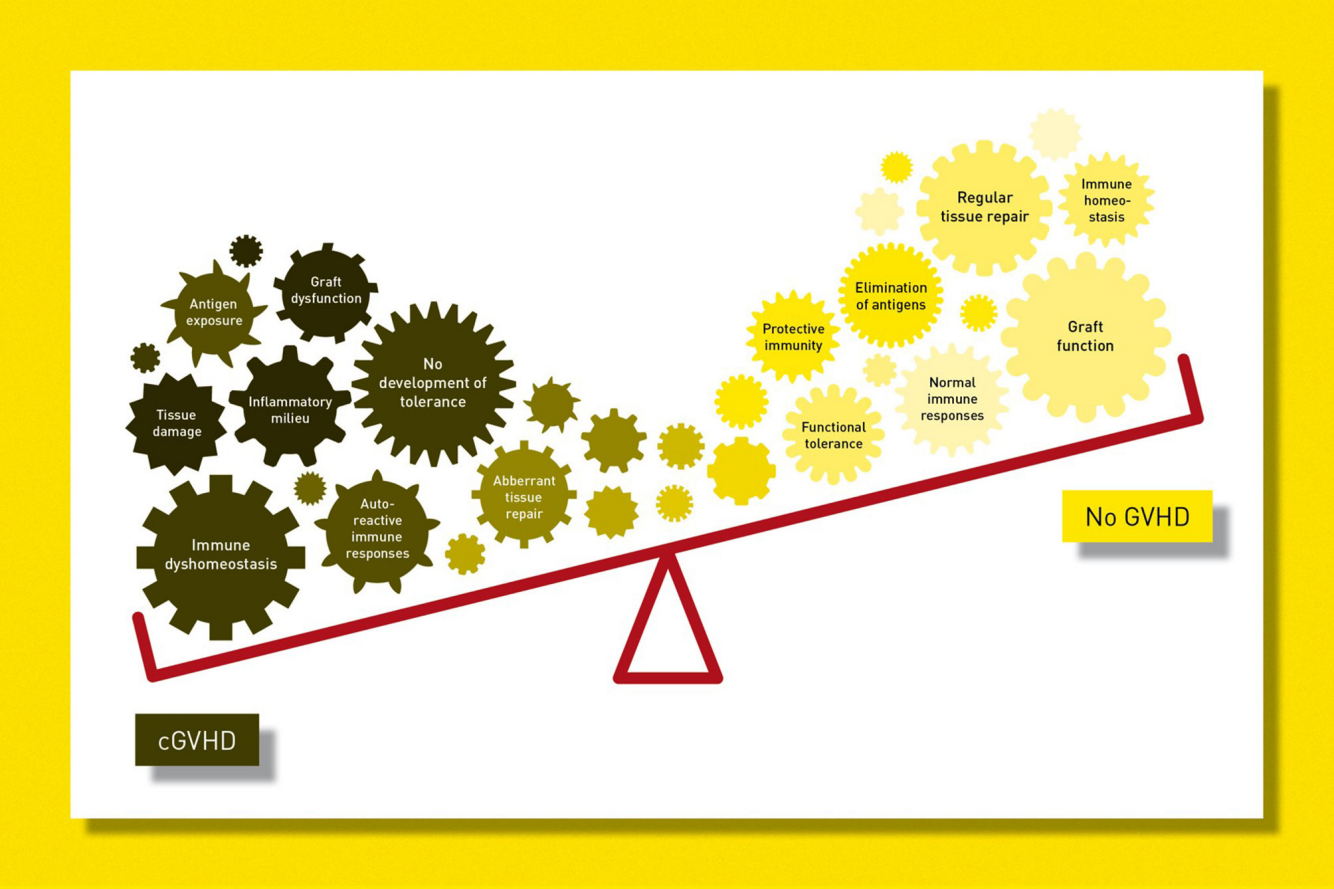
The see-saw of cGvHD.

Figure 3 illustrates the possible insidious onset of cGvHD and the complex interplay with functional correlates. With better insight, the individualised clinical management of paediatric cGvHD and enhanced early intervention may be supported.

#### Risk of ALL Relapse

The association between cGvHD and leukaemic disease control has long been debated and study results are contradictory. A study of Boyiadzis et al. performed in cohort of 7,489 patients with leukaemia including 599 paediatric patients with ALL demonstrated a protective effect of cGvHD against late relapse only for patients with CML (4). Moreover, the presence of cGvHD was associated with significantly higher TRM and worse OS across all diseases studied. Kato et al. described a cohort of 1,030 paediatric patients with ALL in which cGvHD was not found to reduce the risk of post-transplant relapse (3). However, most recently, Yeshurun et al. studied the impact of the GvL effect on survival in 5,215 patients with ALL. In this study were 1,619 paediatric patients and 2,593 adults in CR1/CR2 as well as 1,003 patients with advanced ALL (i.e., CR3 or greater or active disease) (261). The study demonstrated that, both for patients in CR1/CR2 and for patients with advanced ALL, development of cGvHD was associated with a lower risk of relapse.

Thus, it is important to identify the setting in which cGvHD would be most beneficial for leukaemia control by means of developing better cGvHD prevention and therapies in order to improve leukaemia- and event-free survival (4). In addition, it is important to monitor as precisely as possible the post-transplant ALL status of patients during the treatment of cGvHD and to assure early detection of impending relapse and early therapeutic intervention where possible.

##### Monitoring of ALL Status

All subjects with active cGvHD undergoing immunosuppressive treatment should be systematically screened for ALL relapse based on physical examination and results of routine haematological tests, post-transplant haematopoietic chimerism and minimal residual disease (MRD) level. In patients on distinct immunosuppressive treatment for cGvHD, MRD monitoring should be prolonged, especially in those patients who demonstrate a high- or very-high risk score for post-transplant ALL relapse, as proposed by Bader et al. (262). To date, no general recommendation can be given on the best methods or frequency of MRD monitoring in patients with active cGvHD but careful and meticulous execution of the above-mentioned approaches should allow the timely detection of any leukaemia relapses in these patients.

#### cGvHD-Related Immune Impairment and Risk of Infection

Murine studies in combination with biomarker studies have demonstrated a role for T cells as well as B cells in cGvHD. Increased percentages of peripheral naïve CD4^+^CD45RA^+^CD31^−^ Th cells and naïve CD8^+^CD45RA^+^PD-1^+^ cytotoxic T cells as well as activated T cells (CD3^+^CD69^+^) were observed in children with cGvHD compared with patients without cGvHD post HSCT (67). Increased levels of T cells have been observed also in severe compared to moderate cGvHD in children and adolescents (45).

However, GvHD biomarker studies suggest that the hallmark of cGvHD-related immune dysregulation is a profoundly disturbed B-cell profile, with low numbers of transitional memory B cells and lack of differentiation to the switched memory B cell phenotype (56). The most severe cGvHD disease in children and adolescents correlated significantly with a distorted B cell profile consisting of increased CD19^+^CD21^low^ B cells along with an increased CD19^+^CD21^low^ to CD19^+^CD27^+^ B cell ratio (45). Elevated percentages of CD21^low^ B cells have been shown to correlate with the occurrence of severe infections (56). In a third of adult cGvHD patients, this perturbed B cell differentiation leads to significant hypogammaglobulinaemia (57). Conversely, hypergammaglobulinaemia can occur in a subgroup of patients with cGvHD and is associated with the occurrence of allo/autoantibodies, targeting various tissues. Bacterial infections are common in cGvHD and may be the result of dysgammaglobulinaemia aggravated by a degree of functional asplenia (263).

Skin cGvHD was demonstrated to be a specific risk factor for late *Staphylococcus aureus* bacteraemia in a paediatric cohort receiving BM transplants, probably as a result of skin barrier breakthrough (264).

Both aGvHD and cGvHD are risk factors for viral infection and reactivation in paediatric transplant patients, with the highest cumulative incidence for CMV (265, 266). Other pathogens for which risks of infection/reactivation are increased by GvHD include Epstein-Barr virus, adenovirus, BK virus and varicella zoster virus, as well as respiratory infections. Even varicella zoster virus can be fatal in patients with active GvHD on immunosuppressive therapy (267).

A continued risk of invasive fungal infection exists in patients with cGvHD and also paediatric patients who receive high-dose steroids post HSCT (268–270). For patients who develop pulmonary aspergillosis post HSCT yet who continue to need immunosuppressive treatment, the risk of mortality is high, with reports varying from 50–70% (268–270).

##### Infections in Association With Specific Treatment Options

Treatments for cGvHD are often combined making it near impossible to ascertain the risk of infection associated with each separate drug, with the exception of rituximab which in known to cause hypogammaglobulinaemia that directly correlates to increased risks of bacterial and viral infections (271). Most secondary agents are given on a backdrop of some level of steroids. With regards to the newer small molecule therapies, in a study of 22 paediatric patients on ibrutinib for cGvHD, severe bacterial infection (*n* = 2), Epstein-Barr virus reactivations (*n* = 1), and no fungal infections were seen (129). However, data from lymphoma treatment with ibrutinib provide a warning regarding the risk of *Pneumocystis jirovecii* pneumonia and fungal infections (including *Aspergillus*) (272).

Patients with bronchiolitis obliterans syndrome may be at particularly high risk of opportunistic infections when treated with ruxolitinib (273). In an adolescent/adult cohort receiving ruxolitinib/steroid treatment for bronchiolitis obliterans syndrome, a serious infection of grade 2 or higher occurred in 47% of patients, with two thirds of these being fungal infections (273). The myelosuppressive side effects of ruxolitinib result in neutropenia; moreover, its mechanism of action of widely blocking innate and specific immune intracellular cytokine signalling makes cGvHD patients receiving this therapy more prone to developing all types of infection (especially viral infections but also candida, fungal and mycobacterial infections) (186, 273, 274).

Of all treatments for cGvHD discussed above, ECP appears to be associated with the lowest rate of infection (275).

#### cGvHD-Related Organ Toxicity and the Risk of Complications and Late Effects

Both the highly inflammatory state and immune dysregulation seen in cGvHD and the side effects of medications can damage organ systems. This can lead to new organ dysfunctions that will require new medical interventions over time. The long-term toxicities and complications of paediatric cGvHD are the result of a complex interplay of symptoms and dysfunctions which impact on physical functioning and quality of life, with inferior outcomes associated with severe cGvHD (2). A summary of the main treatment-related toxicities is shown in Table 4.

The long-term consequences of these late complications in children are possibly: (i) an impairment of future developmental potential within a growing organ system (276), and (ii) an increase in morbidity of chronic health conditions occurring throughout life (277). The occurrence and patterns of late toxicity and complications associated with cGvHD and its treatment depend on the intensity of conditioning (especially TBI-based conditioning) and patient age at transplant (278, 279) and at beginning of complications. These complications contribute to late comorbidities (280). Nearly any organ might be affected by them, including the development of secondary malignancies (15, 281, 282). As the life expectancy of paediatric patients post HSCT continues to increase, these chronic health conditions are a significant burden in the population of transplant survivors.

#### Proposal for the Personalised Management of Paediatric CGvHD From the Clinicians' Viewpoint

##### A Checklist for Individualised Risk Evaluation With Aggregated Considerations

**Figure 4:**
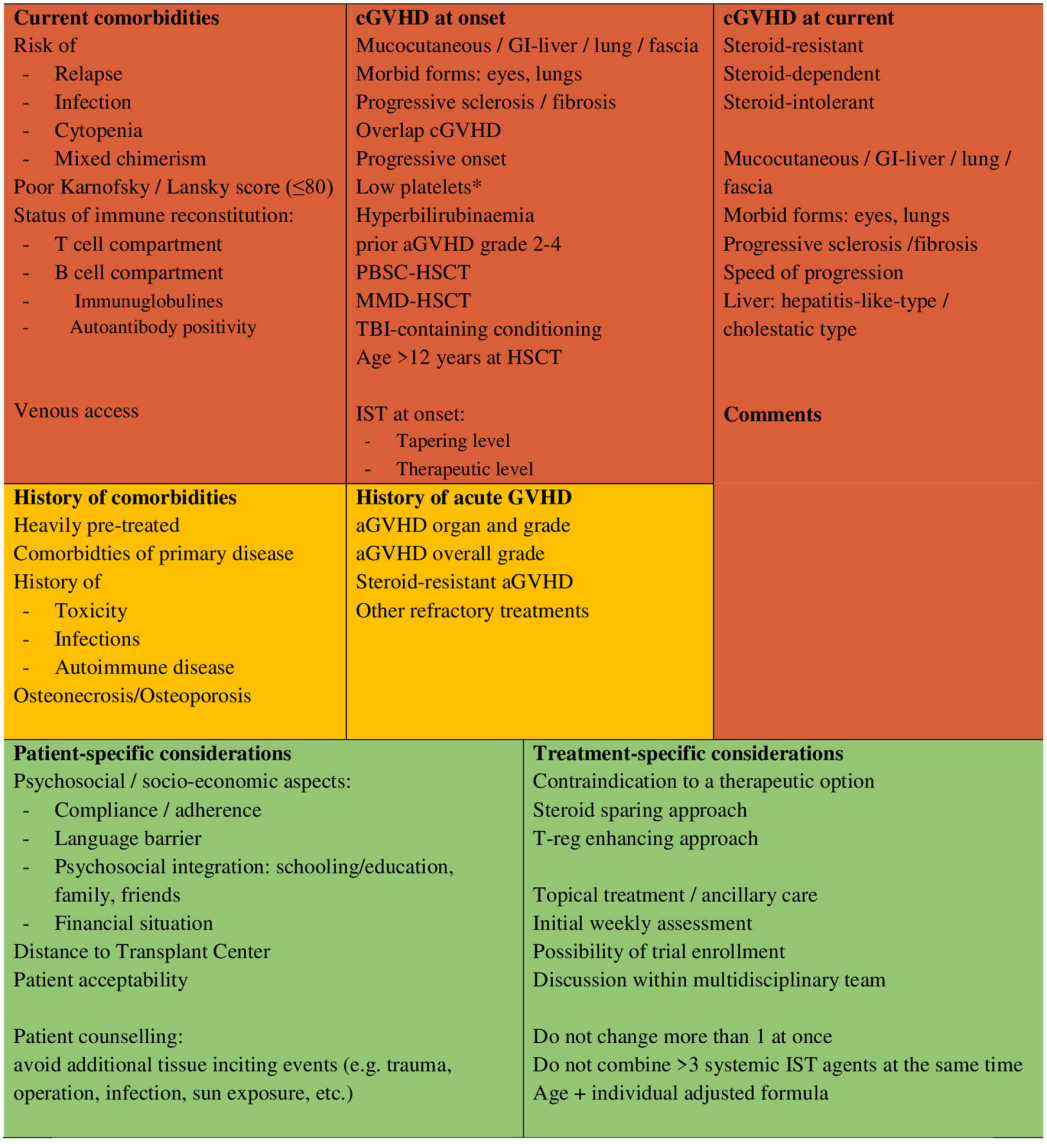
Individualised risk assessment and aggregated considerations (cheque as appropriate). *Platelets < 100 *Gil*. GI, gastrointestinal tract; PBSC, peripheral blood stem cells; HSCT, hematopoietic stem cell transplantation; MMD, mismatched donor; TBI, total body irradiation; IST, immunosuppressive therapy.

As intervention and treatment decisions in daily clinical practice are both clinician and patient specific, we summarise here the most important aspects including evaluation of the individual's risk and prognostic indicators as well as an assessment of aggregated considerations. The evaluation of individual risk and prognostic indicators covers details of GvHD and immune reconstitution, the primary disease and relapse risk, comorbidities and infectious complications. Aggregated considerations cover details of the patient's individual psychosocial and socio-economic circumstances and take into account their personal tolerance and preferences. To capture all these details, we created a comprehensive colour-coded checklist for routine clinical use (Figure 4). This walk-through checklist will provide the clinician with a summary of the patient's status and should be used at baseline and each timepoint of clinical evaluation, helping the clinician to identify various co-existing aspects at one glance.

The rationale behind this approach is to better identify the appropriate time point for the most appropriate treatment approach in a patient-centred manner, keeping in mind that prevention of severe cGvHD is of utmost importance (11).

##### A Treatment Algorithm for Paediatric cGvHD Patients at High Risk of Relapse

**Figure 5:**
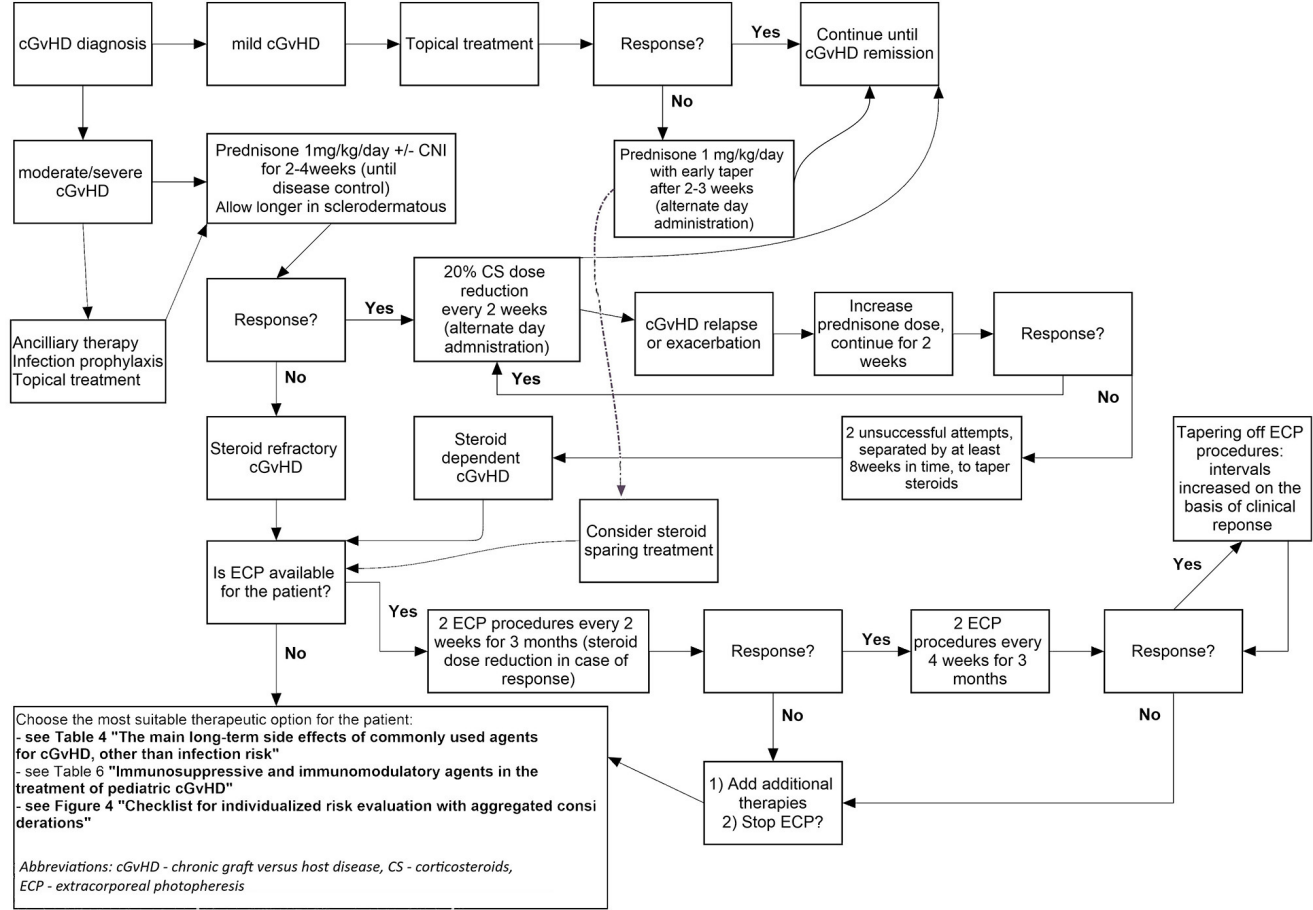
Treatment algorithm for paediatric cGvHD patients at high risk of relapse.

The desirable therapeutic approach to managing paediatric cGvHD patients at high risk of relapse would consist of a safe treatment with minimum short-and long-term adverse events, embedded within an evidence-based protocol and supported by reliable predictors of response. Currently, data are not available to support such a therapeutic approach and it is likely that therapeutic interventions will not benefit all patients. Therefore, we propose a treatment algorithm to inform the personalised management plans of high-risk patients, which we developed based on the literature and joint clinical experience (Figure 5). The algorithm uses representative paediatric patients with cGvHD following HSCT for ALL who are at high risk of relapse.

We recommend that, for patients at high risk of relapse, clinicians use both our checklist for risk evaluation and our treatment algorithm to inform personalised management plans. Given the variety of organ-specific cGvHD manifestations and comorbidities that patients may present with and various patient-specific considerations, we recommend that each patient's cGvHD management plan is discussed within a multidisciplinary team.

## Discussion

Similarly to adults, cGVHD in children presents as a complex multi-system disease with high interindividual heterogeneity and with a distinctly inconsistent intraindividual disease course. Given the debilitating consequences and the potentially life-threatening nature of cGvHD, recognition of the earliest signs and symptoms and an early timepoint of intervention are of utmost importance. The prevention of severe and highly morbid forms of paediatric cGvHD is a main goal of management (11–13). Within the limits of this review, current knowledge has been summarised and gaps in knowledge have been identified. To facilitate the early recognition of this complex disease for the clinician, we have put forth a theory of a holistic view of paediatric cGvHD and its associated manifestations.

Improved understanding of the immunobiology of cGvHD, more precise diagnosis by the application of various biomarkers, and the identification of new therapeutic targets is required. Beside this, the treatment choices of paediatric cGvHD—and especially SR-cGvHD—remain clinician and patient specific in daily clinical practice. As no standardised recommendations exist regarding when and how to modify treatment, and in light of a risk of relapse, infection and comorbidity, we developed an individualised cGvHD management plan aiming for the titration of immunosuppressive treatment according the current status of the patient.

We have proposed a walk-through checklist for individualised risk evaluation with aggregated considerations to provide the clinician with a summary of the patient's status. Ideally this checklist should be used at baseline and each timepoint of clinical evaluation, helping the clinician to identify various co-existing aspects at one glance during clinical follow up.

Moreover, using representative cases of paediatric cGvHD after HSCT for ALL, we have proposed a treatment algorithm for those patients at high risk of relapse. ECP with its GvL sparing and immunomodulatory effect and no serious side effects seems beneficial for this patient group, although standardised recommendations regarding the ECP treatment schedule in paediatric patients are lacking. The mode of vascular access, the benefit of earlier introduction of ECP after paediatric HSCT, and the broader use of mini ECP remain areas where further research is warranted.

Our proposed approach is mainly based on the literature and expert opinions and will require confirmation *via* well-designed studies. In lieu of the evidence-based data needed to inform individualised cGvHD management in paediatric patients, we hope our proposed approach that focuses on patients' individual needs will help clinicians to improve their clinical management of cGvHD.

Evidence-based data from ongoing studies are eagerly awaited, especially regarding the recently FDA-approved treatment ruxolitinib, allowing more targeted treatment. The possible risk of infectious complications with ruxolitinib must be taken into account, again pointing out a possible advantage of ECP in this regard.

In conclusion, as a complex multiorgan disease with manifold pathogenetic pathways and the presentation of multiple manifestations over time, paediatric cGvHD requires optimal patient-adjusted management with flexible regimens chosen for specific clinical findings according to each patient's risk profile and circumstances.

## Data Availability Statement

The original contributions presented in the study are included in the article/Supplementary Material, further inquiries can be directed to the corresponding author.

## Author Contributions

AS-S, CL, TS, JW, J-HD, HB, and AL contributed to conception, design of the study, and wrote the first draft of the manuscript. AS-S, CL, TS, JW, J-HD, HB, AG, and AL wrote sections of the manuscript. All authors contributed to manuscript revision, read, and approved the submitted version.

## Funding

This study received funding from the St. Anna Children's Cancer Research Institute, Vienna, Austria. The funders were not involved in the study design, collection, analysis, interpretation of data, the writing of this article, or the decision to submit it for publication.

## Conflict of Interest

The authors declare that the research was conducted in the absence of any commercial or financial relationships that could be construed as a potential conflict of interest.

## Publisher's Note

All claims expressed in this article are solely those of the authors and do not necessarily represent those of their affiliated organizations, or those of the publisher, the editors and the reviewers. Any product that may be evaluated in this article, or claim that may be made by its manufacturer, is not guaranteed or endorsed by the publisher.
