# Supplementary material for: Management of Chronic Graft-vs.-Host Disease in Children and Adolescents With ALL: Present Status and Model for a Personalised Management Plan
Source: Front Pediatr. 2022 Feb 18;10:808103. doi: 10.3389/fped.2022.808103 (PMC8894895; doi:10.3389/fped.2022.808103)

Pediatric adaption of NIH 2014 cGVHD diagnosis/staging for clinical practice
German-Austrian-Swiss GVHD Consortium

completed by: date patient name

► please score/check the worst manifestation

► diagnostic features are marked **bold**

classification:

□ feat. of acute GVHD

□ feat.of classic cGVHD

□ both

onset type at diagnosis:

□ de novo

□ quiescent

□ progressive

height: ______________

weight: ______________

**symptoms/features**	**Score 0**	**Score1**	**Score 2**	**Score 3**
**KPS/LPS:** %	□ asymptomatic and fully active (KPS/LPS 100%)	□ sympt., fully amb., restricted only in physically strenous activity (KPS/LPS 80-90%)	□ sympt.,amb., capable of self-care, >50% of waking hours out of bed (KPS/LPS 60-70%)	□ sympt., limited self-care >50% of waking hours in bed (KPS/LPS < 60%)

SKIN

Feat. scored by BSA:	no BSA involved	1-18% BSA	19-50% BSA	> 50% BSA
□ maculopapular rash/erythema	□	□	□	□
□ **lichen planus-like features**	□	□	□	□
□ **sclerotic features:**	□	□	□	□
□ **lichen sclerosus-like**	□	□	□	□
□ papulosquamous lesions	□	□	□	□
□ ichthyosis	□	□	□	□
□ keratosis pilaris-like GVHD	□	□	□	□

Feat. not scored by BSA:

□ hyperpigmentation

□ hypopigmentation/ depigmentation

□ **poikiloderma**

□ severe pruritus

□ hair involvement

□ nail involvement

□ sweat impairment

□ abnormality present but explained entirely by non-GVHD cause (specify):

□ abnormality thought to represent GVHD **PLUS** other causes (specify):

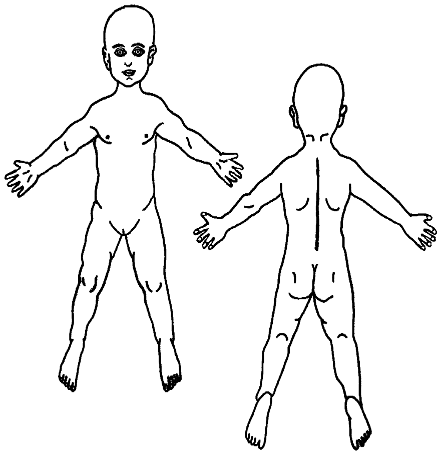

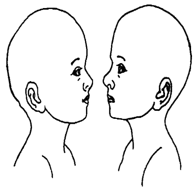

%BSA:

child: head front/back 9 / 9
back 18, chest 18,
arm left 9, arm right 9
leg left 13,5, leg right 13,5

adult: head front/back 4,5 / 4,5
back 18, chest 18
arm left 9, arm right 9
leg left 18, leg right 18

palm: 1,5

**sclerotic features:**	□ no sclerotic features		□ superficial sclerotic features "not hidebound" (able to pinch)	□ deep sclerotic features<br>□ "hidebound" (unable to pinch)<br>□ impaired mobility<br>□ ulceration

MOUTH

□ erythema<br>□ **lichen planus-like features**<br>□ hyperkeratot. plaques<br>□ mucoceles □ pseudomembranes<br>□ ulcers □ mucosal atrophy<br>□ dryness □ pain	□ no symptoms	□ mild sympt with disease signs but not limiting oral intake significantly	□ moderate sympt. with disease signs with partial limitation of oral intake	□ severe sympt. with disease signs on examination with major limitation of oral intake

□ abnormality present but explained entirely by non-GVHD cause (specify):

□ abnormality thought to represent GVHD **PLUS** other causes (specify):

symptoms/features	Score 0	Score1	Score 2	Score 3

EYES

□ keratokonjunktivitis sicca (KCS)<br>□ confirmed by opthalmologist<br>□ dryness □ pain<br>□ photophobia □ blepharitis<br>□ pseudomembranes □ ulcers	□ no symptoms	□ mild dry eye sympt. not affecting ADL (requirement of lubricant eye drops ≤ 3 x per day)	□ moderate dry eye sympt. partially affecting ADL (lubricant eye drops >3 x/d or punctual plugs) without new vision impairement due to KCS	□ severe dry eye sympt. significantly affecting ADL (special eyeware to relieve pain) or unable to work because of ocular sympt or loss of vision due to KCS

□ abnormality present but explained entirely by non-GVHD cause (specify):
□ abnormality thought to represent GVHD **PLUS** other causes (specify):

GI TRACT

□ **esophageal web/ prox stricture or ring**<br>□ dysphagia □ abdominal pain<br>□ anorexia □ failure to thrive<br>□ nausea □ vomiting<br>□ diarrhea □ weight loss ≥ 5%	□ no symptoms	□ symptoms without significant weight loss (5%)	□ sympt. associated with mild to moderate weight loss (5-15%) or moderate diarrhea without significant interference with daily living	□ symptoms associated with significant weight loss (> 15%) requires nutritional supplement for most calorie needs or esophageal dilatation or severe diarrhea with signif. Interference with daily living

□ abnormality present but explained entirely by non-GVHD cause (specify):
□ abnormality thought to represent GVHD **PLUS** other causes (specify):

LIVER

□ hepatitic pattern	□ normal total bili and ALT or AP < 3 ULN	normal total bili<br>□ with ALT ≥ 3-5x ULN<br>□ or AP ≥ 3 x ULN	elevated total bili<br>□ but ≤ 3 mg/dl or<br>□ ALT > 5 ULN	□ elevated total bili > 3 mg/dl

□ abnormality present but explained entirely by non-GVHD cause (specify):
□ abnormality thought to represent GVHD **PLUS** other causes (specify):

LUNGS

**FEV1**:______% MEF25:_____%<br>FVC:_______% MEF50:_____%<br>DLCO:______% MEF75:_____%<br>RV: ______ □ RV/TLC >120%<br>CT:	□ no symptoms<br>□FEV1 ≥ 80%	□ mild symptoms (shortness of breath after climbing one flight of steps)<br>□FEV1 60-79%	□ moderate symptoms (shortness of breath after walking on flat ground)<br>□FEV1 40-59%	□ severe symptoms (shortness of breath at rest; requiring O2)<br>□FEV1 ≤ 39%

□ pulmonary function test not performed
□ abnormality present but explained entirely by non-GVHD cause (specify):
□ abnormality thought to represent GVHD **PLUS** other causes (specify):

JOINTS AND FASCIA

ped P-ROM score (see below)<br>□ edema □ fasciitis<br>□ muscle cramps □ athralgia	□ no symptoms	□ mild tightness, normal or mild ↓ of range of motion (ROM) not affecting ADL	□ tightness or joint contractures, fasciitis, moderate ↓ of ROM, mild - moderate ↓ of ADL	□ contractures, fasciitis significant ↓ of ROM, significant ↓ of ADL

□ abnormality present but explained entirely by non-GVHD cause (specify):
□ abnormality thought to represent GVHD **PLUS** other causes (specify):

GENITAL TRACT

□ erosions, fissures<br>□ **lichen planus-like features**<br>□ **lichen sclerosus-like features**<br>□ **labial/ vaginal scarring** □ **phimosis**	□ no signs	□ mild signs	□ moderate signs	□ severe signs with or without symptoms

□ **currently sexually active** **coitarche:____years**

□ **hormonal status: hypogonadism** □ **signs of vaginal hypoestrogenization**

□ abnormality present but explained entirely by non-GVHD cause (specify):
□ abnormality thought to represent GVHD **PLUS** other causes (specify):

Overall GVHD severity

□ no cGVHD		maximum individual score
□ mild:	max. score of 1 in any affected organ, max. 2 organs affected, no lung involvement	total score (sum)
□ moderate:	≥3 organ with max score 1 or max. score of 2 in any affected organ, lung score max 1	average score (total score/24)
□ severe:	score 3 in any affected organ, lung score 2-3	number of affected organs

Other indicators, clinical features or complications related to cGVHD			biopsy: Yes □ No □
check all that apply and assign a severity score (0-3) based on functional impact			date:
□ ascites (serositis)	□ myasthenia gravis	□ diabetes	organ:
□ pericardial effusion	□ peripheral nervous manifestations	□ eosinophilia >500 /ul	
□ pleural effusion	□ central nervous manifestations	□ platelets <100 000/ul	GVHD confirmed? Yes □ No □
□ nephrotic syndrome	□ polymyositis	□ hypo/hyperglobulinemia	suspicion
□ others (specify)	□ weight loss >5% without GI symptoms	□ auto-antibodies	
		□ immune thyroiditis	

Change from prior evaluation:
□ improved □ stable □ worse □ comment:___________________

Intensity of current immunosuppression
- □ None
- □ Mild (single agent prednisone<0.5 mg/kg/day)
- □ Moderate (prednisone≥0.5 mg/kg/day and/or any single agent/modality)
- □ High (2 or more agents/modalities ± prednisone≥0.5 mg/kg/day)

Therapeutic intent at the time of clinic visit
- □ Decision to decrease systemic therapy because cGVHD is better
- □ Decision is to not change current systemic therapy because cGVHD is stable
- □ Decision is to increase systemic therapy because cGVHD is worse
- □ Alter systemic therapy due to its toxicity
- □ Substitute systemic therapy due to lack of response
- □ Withdraw systemic therapy due to lack of response
- □ Not applicable

Clinician's impression of activity
- □ Inactive, off systemic therapy or topical immunosuppression
- □ Inactive, on systemic therapy or topical immunosuppression
- □ Active, irrespective of the level of current therapy
- □ Highly active, irrespective of the level of current therapy

Pediatric Photographic Range of Motion (adapted ped P-ROM)

please mark appropriate number

shoulder: 1 (worst) 2 3 4 5 (normal)

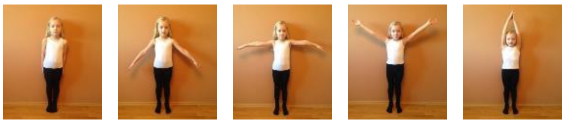

ellbow: 1 (worst) 2 3 4 (normal)

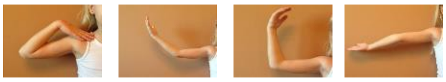

wrist / finger: 1 (worst) 2 3 4 (normal)

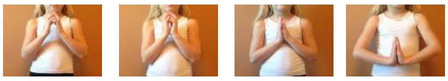

global flexion: 1 (worst) 2 3 4 (normal)

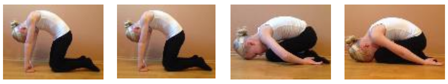

ankle: 1 (worst) 2 3 (normal)

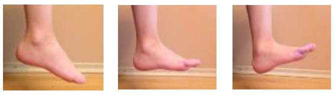

Supplement: Supplementary file 1 [file Data_Sheet_1.PDF]
